# Prognostic value of serum phosphate levels in sepsis: a systematic review and meta-analysis

**DOI:** 10.7717/peerj.16241

**Published:** 2023-10-13

**Authors:** Shengfeng Wei, Yunhan Li, Chunhua Zhang, Xiangjian Guo, Xinmeng Liang, Yanmei Huang, Fan Zhang, Jihong Li, Qiangqiang Liu

**Affiliations:** Department of Emergency Medicine, The First Affiliated Hospital, Sun Yat-sen University, Guangzhou, China

**Keywords:** Sepsis, Phosphate, Hyperphosphatemia, Hypophosphatemia, Meta-analysis, Review

## Abstract

**Background:**

There remain controversies over the conclusion of different serum phosphate levels as prognostic predictors of sepsis patients. As such, this study investigated the association between different serum phosphate and the prognosis of sepsis.

**Methods:**

Data from PubMed, Embase, Cochrane Library, and Web of Science were systematically retrieved from the inception of databases to June 1, 2023 and independently screened and extracted by two authors. Binary variables in the study were estimated as relative risk ratio (RR) and 95% confidence interval (CI), and continuous variables were estimated as mean and standard deviation. The Newcastle-Ottawa Scale (NOS) was employed to evaluate the quality of the included studies, and subgroup analysis and sensitivity analysis were performed for all outcomes to explore the sources of heterogeneity.

**Results:**

Ten studies were included in this study including 38,320 patients with sepsis or septic shock. Against normal serum phosphate levels, a high serum phosphate level was associated with an elevated all-cause mortality risk (RR = 1.46; 95% CI [1.22–1.74]; *P* = 0.000) and prolonged Intensive Care Unit (ICU) length of stay (LOS) (WMD = 0.63; 95% CI [0.27–0.98]; *P* = 0.001). However, there was no significant difference in the in-hospital LOS (WMD = 0.22; 95% CI [−0.61–1.05]; *P* = 0.609). A low serum phosphate level was not significantly associated with the all-cause mortality risk (RR = 0.97; 95% CI [0.86–1.09]; *P* = 0.588), ICU LOS (WMD = −0.23; 95% CI [−0.75–0.29]; *P* = 0.394) and in-hospital LOS (WMD = −0.62; 95% CI [−1.72–0.49]; *P* = 0.274).

**Conclusion:**

Sepsis patients with high serum phosphate levels before therapeutic interventions were associated with a significant increase in the all-cause mortality risk, prolonged ICU LOS, and no significant difference in in-hospital LOS. Sepsis patients with low serum phosphate levels before interventions may have a reduced risk of all-cause mortality, shorter ICU LOS, and in-hospital LOS, but the results were not statistically significant.

## Introduction

Sepsis is a common yet complex disease with high incidence and has been one of the leading causes of death worldwide (Cecconi et al., 2018). A report by Rudd et al. (2020) (Global Burden of Disease Study) found that sepsis-associated mortality was approximately 22.5%, accounting for 19.7% of global deaths. High mortality leads to a heavy economic burden. The annual treatment expense for sepsis in Japan is up to 4.38 billion US dollars, with an increasing trend (Oami et al., 2022). Since 2017, the World Health Assembly and World Health Organization (WHO) have placed sepsis high on the list of global health challenges and committed to its prevention, diagnosis, and management (Goodman & Brett, 2017). As the research for sepsis advances, a number of prognostic predictors associated with sepsis, such as procalcitonin, C-reactive protein, and lactic acid, have been proposed (Barichello et al., 2022; Goh et al., 2021). Despite updates in clinical guidelines, the reliability of predictors for the early diagnosis of sepsis is limited by its overlapping symptoms with other diseases (Huang et al., 2023). Therefore, new markers for timely diagnosis and treatment of sepsis are still required.

Phosphate, as an essential element in the human body, is conducive to maintaining muscle contraction and cell integrity, delivering nerve stimulation, and keeping normal organ functions. Several studies have shown that phosphate is associated with clinical outcomes of various diseases, including malignancy, cardiovascular, and kidney diseases (Minisola et al., 2017; Vervloet et al., 2017). However, researchers have contradicting opinions on its role in predicting sepsis. Shor et al. (2006) found low phosphate levels to be an independent predictor of death in sepsis patients. Nonetheless, Schwartz et al. (2002) believed that low phosphate level was not associated with sepsis-related mortality, whereas the mortality in patients with high phosphate levels was significantly high (Black et al., 2020). Besides, a recent clinical trial task (NCT04519762) sponsored by Ain Shams University is still investigating the effect of the phosphate level on the outcome of sepsis patients (NCT, 2020). Therefore, the association between serum phosphate disorder and the clinical outcome of sepsis remains controversial internationally, and there is currently no relevant meta-analysis. For this reason, this study was to summarize relevant studies, clarify different serum phosphate levels on sepsis, comprehensively explore its relationship with the prognosis of sepsis, and provide a clinical basis for the early warning and intervention of sepsis.

## Materials and Methods

This systematic review and meta-analysis complied with the Preferred Reporting Items for Systematic Reviews and Meta-Analyses (PRISMA) Statement (Page et al., 2020). The study protocol has been registered on PROSPERO, an international prospective registry platform for systematic reviews (CRD42023425450), and its entire content is available on https://www.crd.york.ac.uk/PROSPERO/display_record.php?RecordID=425450.

### Search strategy

Two authors (Sf-W and Yh-L) independently and systematically retrieved data from PubMed, Embase, Cochrane Library, and Web of Science from the inception of database to June 1, 2023. The retrieval terms included Sepsis, Mortality, Phosphate, Hyperphosphatemia, and Hypophosphatemia, and medical subject words and keywords were used for combined retrieval. The detailed retrieval strategy is provided in File S1. All studies were screened using EndNote X9.

### Inclusion and exclusion criteria

Studies eligible for inclusion were selected according to the PICOS principles:

(1) Patients: Sepsis patients aged ≥18;

(2) Exposure: Hyperphosphatemia or hypophosphatemia before sepsis intervention (as defined by the original studies);

(3) Control group: Normal phosphate level before sepsis intervention;

(4) Outcome: All-cause mortality, ICU LOS (length of stay), and in-hospital LOS;

(5) Study type: Case-control, cohort, or cross-sectional study.

Studies meeting any of the following criteria were excluded:

(1) No clear report of defined hyperphosphatemia or hypophosphatemia;

(2) Animal experiments;

(3) Meta-analysis or review, case reports, letters, and meeting abstracts;

(4) No prognostic outcomes or sufficient data.

### Data extraction and quality assessment

All retrieved studies were imported into EndNote, and duplicate publications were removed. Then two authors (Sf-W and Yh-L) screened the remaining studies by title, abstract, and full text. The following data were then extracted independently from the included studies: Author, Year, Country, Design, Sample size, Age, Gender, Adjusted variables, Follow-up, and Outcomes.

The authors (Sf-W and Yh-L) independently used the Newcastle-Ottawa Scale (NOS) to assess the quality of the included nonrandomized studies in this meta-analysis (Stang, 2010). The NOS contains eight items in three dimensions: Selection, Comparability, and Outcomes. For each item, the scale provides a range of answer options. A star rating system is used for a semi-quantitative assessment of the study quality. Studies with the highest quality may be given one star per item, but two stars are allowed for the item related to comparability. The total NOS score ranges from 0–9 stars, with a study scoring greater than 6 considered high-quality and a study scoring less than or equal to 6 considered low-quality. Dissents in the data extraction and quality evaluation process, if any, would be discussed and resolved with a third investigator.

### Statistical analysis

Meta-analysis was performed for the outcomes from all included studies. Binary variable outcomes were expressed as Relative Risk (RR) with 95% confidence interval (CI), and the continuous variables were expressed by mean and standard deviation. The heterogeneity among studies was analyzed using the Q test and the I^2^ test. If I^2^ is ≥50% and *P*-value is <0.1, there is significant heterogeneity, and the random-effects model (REM) would be used. Otherwise, the fixed effects model (FEM) will be used. In the case of large heterogeneity, a subgroup analysis was conducted through the single-factor or multi-factor analysis method to explore the source of heterogeneity. Sensitivity analysis was carried out to explore the stability of the results by eliminating the literature one by one, and the publication bias was assessed for all the outcomes by Egger’s test and Begg’s test. A *P*-value ≤ 0.05 indicates a statistically significant difference. Stata SE 15.1 (Stata Corp. LD, College Station, TX, USA) was used for the above analyses.

## Results

### Study selection

**Figure 1:**
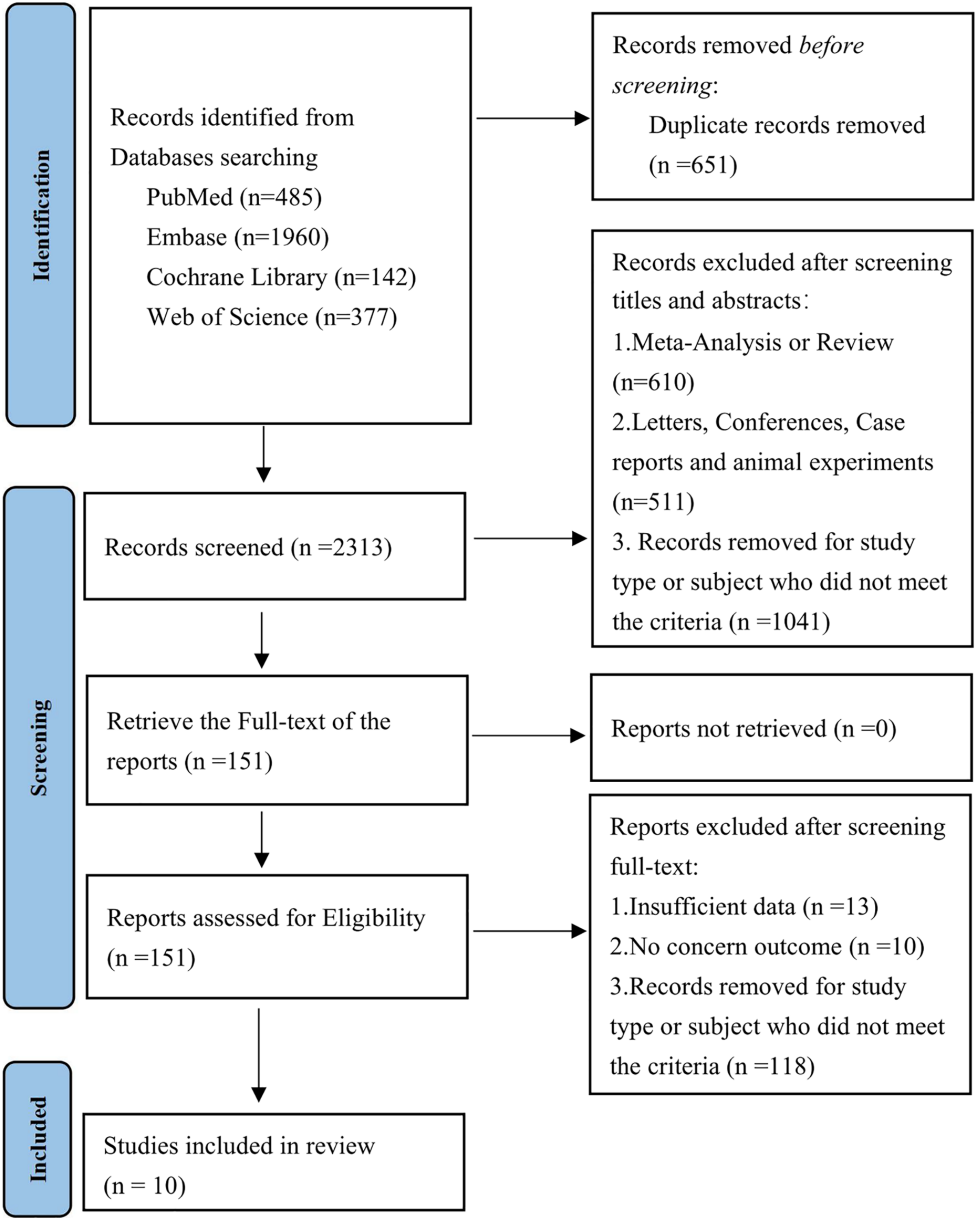
PRISMA flowchart of selection of studies for inclusion in this meta-analysis.

A total of 2,964 articles were retrieved from databases, of which 651 were duplicate publications. A total of 151 were preliminarily eligible by title and abstract. After a full-text review of the 151 studies, 141 were excluded. Finally, 10 studies (Jung et al., 2016; Al Meshari et al., 2019; Jang et al., 2020; Miller et al., 2020; Wang et al., 2020, 2021; Cao et al., 2021; Guo et al., 2022; Li, Shen & Han, 2022; Xu et al., 2023) were included in the meta-analysis (Fig. 1).

### Study characteristics and methodological quality

A total of 38,320 patients with sepsis or septic shock were involved in the included studies, with a minimum sample size of 197 and a maximum sample size of 11,658. The main characteristics of these patients are summarized in Table 1. The included studies were published between 2016 and 2023, with one study (Cao et al., 2021) being prospective and the rest retrospective. Of the 10 studies, nine were from Asia and one (Miller et al., 2020) was from North America. The mean age of the study participants ranged from 60.1 ± 13.83–74 ± 11.85. The follow-up duration ranged from 28 days to 11 years. The proportion of male patients was about 55.7%. Regression analysis was used in seven of the ten studies to adjust the effect of confounding factors on mortality, and the adjusted factors included at least age, gender, Body Mass Index (BMI), Sequential Organ Failure Assessment (SOFA) score, and comorbidities. The details are provided in Table 1. Six of the ten studies reported Odds Ratio (OR) with 95% CI for mortality between abnormal and normal phosphate levels, which were converted into RR with 95% CI for primary outcomes to investigate the ratio between the two. The NOS quality scores of the included studies ranged from 8–9, and all ten studies were considered high-quality, as shown in File S1.

**Table 1:** Characteristics of all included studies.

Authors	Year	Country	Design	Sample size	Age	Gender(M/F)	Adjusted variables	Follow up	Outcomes	NOS
Jung et al.	2016	South Korea	Retrospective	210	62.2 ± 12.90	136/74	Age, gender, BMI at ICU admission, CCI, SOFA score, eGFR (0 h)	28 days	28-day mortality	8
Al Meshari et al.	2019	Saudi Arabia	Retrospective	1,422	66.1 ± 18.92	826/ 596	Age, APACHE II, sex, serum creatinine	3 years	In-hospital mortality, ICU LOS, hospital LOS	9
Jang et al.	2020	South Korea	Retrospective	3,034	74.0 ± 11.85	1,745/1,289	NONE	28 days	28-day mortality, ICU LOS, hospital LOS	9
Miller et al.	2020	United States	Retrospective	197	60.1 ± 13.83	94/103	NONE	28 days	28-day mortality	8
Wang et al.	2020	South Korea	Retrospective	796	63.5 ± 14.18	494/302	Age; sex; body mass index; systolic blood pressure; diastolic blood pressure; myocardial infarction; congestive heart failure; cerebrovascular disease; peripheral vascular disease; diabetes mellitus; hypertension; chronic obstructive pulmonary disease; continuous renal replacement therapy indication; Charlson comorbidity index; albumin;APACHE II score; sequential organ failure assessment score; C-reactive protein; glomerular filtration rate; serum creatinine; mechanical ventilation at CRRT initiation; white blood cell count; haemoglobin; blood urea nitrogen; K^+^; HCO3^–^	28 days	28-day mortality	9
Cao et al.	2021	China	Prospective	294	67.0 ± 15.56	185/109	NONE	90 days	All-cause mortality	9
Wang et al.	2021	Israel	Retrospective	4,767	65.1 ± 17.74	2,634/2,133	Age, male, ethnicity, ventilation, vasopressin use, comorbidities, laboratory data, SOFA	90 days	In-hospital mortality, ICU LOS, hospital LOS	9
Guo et al.	2022	Israel	Retrospective	6,251	66.4 ± 15.62	3,533/2,718	Age; gender; HR; SBP; DBP; RR; AG; ALT, AST,bicarbonate, INR, magnesium, total bilirubin; total calcium; creatinine; chloride; hematocrit; hemoglobin; lactate; MCV; MCH; MCHC; PLT; PT; TT; potassium; RBC; RDW; urea nitrogen; WBC; sodium; renal disease; CAD; diabetes; hypertension	30 days	30-day mortality, ICU LOS, hospital LOS	9
Li et al.	2022	Israel	Retrospective	11,658	67.4 ± 16.00	6,062/5,596	Age, gender, SOFA scores, weight, vasopressor use, hypertension, CAD, CKD, AKI,WBC count, serum creatinine level, blood lactate level, respiratory infection, urinary infection, and bloodstream infection	11 years	In-hospital mortality	8
Xu et al.	2023	Israel	Retrospective	9,691	65.21 ± 16.61	5,623/4,068	Age, gender, weight, all ICU comorbidities, SOFA score	28 days	28-day mortality, ICU LOS, hospital LOS	9

**Note:**

OR, odds ratio; RR, relative risk; HR, hazard ratio; NOS, the Newcastle-Ottawa Scale; LOS, length of stay; BMI, body mass index; CCI, Charlson comorbidity index; eGFR, estimated glomerular filtration rate; SOFA score, sequential organ failure assessment score; APACHE, acute physiology and chronic health evaluation; CRRT, continuous renal replacement therapy; HR, heart rate(Adjusted variables); SBP, systolic blood pressure; DBP, diastolic blood pressure; RR, respiratory rate (Adjusted variables); AG, anion gap; ALT, alanine aminotransferase; AST, aspartate aminotransferase; INR, international normalized ratio; MCV, mean corpuscular volume; MCH, mean corpusular hemoglobin; MCHC, mean corpusular hemoglobin concentration; PLT, platelet; PT, prothrombin time; TT, thrombin time; RBC, red blood cells; RDW, red blood cell distribution width; WBC, white blood cell; CAD, coronary artery disease; CKD, chronic kidney disease; AKI, acute kidney disease.

### Primary outcome

#### All-cause mortality

##### Hyperphosphatemia vs Normophosphatemia

**Figure 2:**
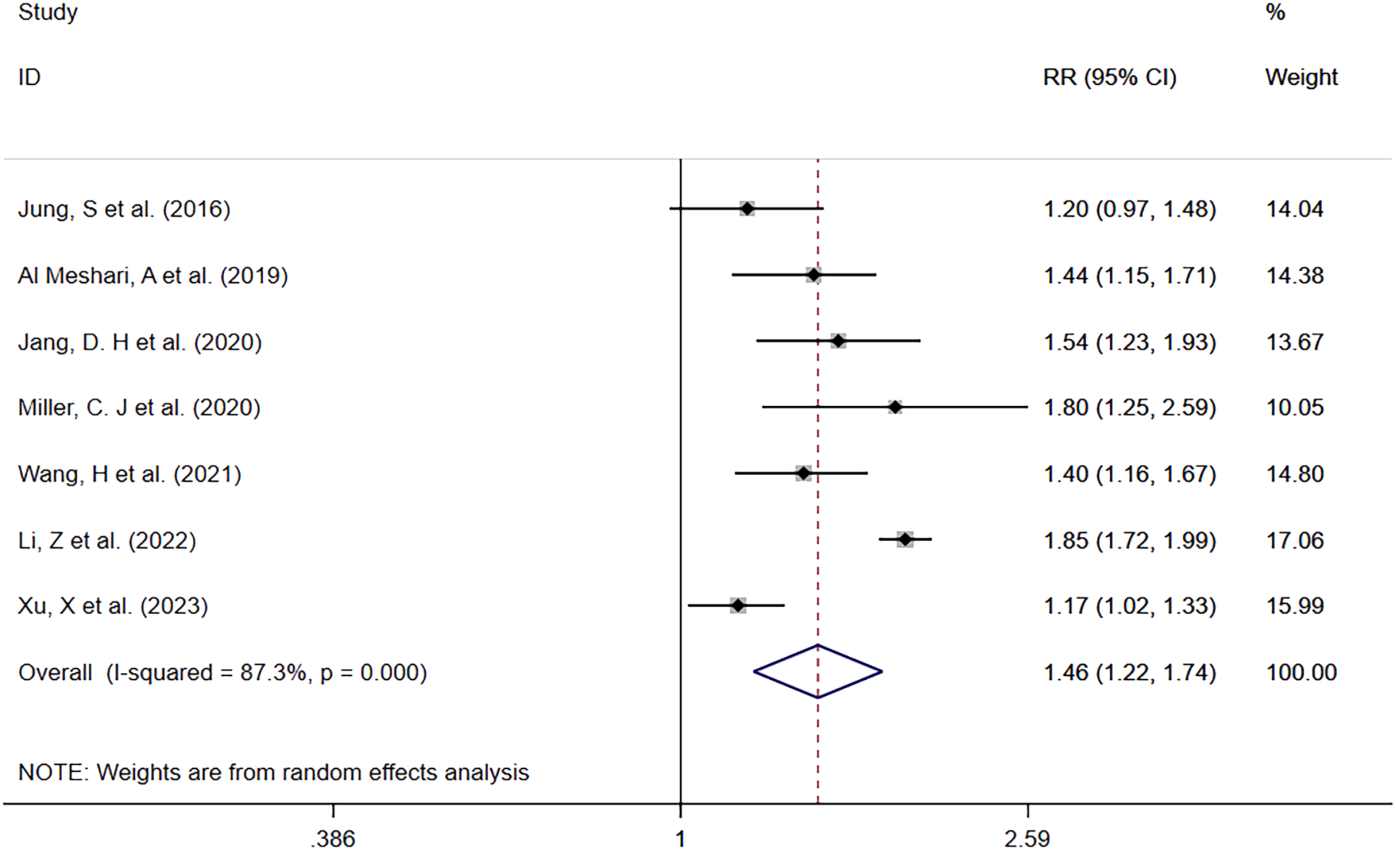
Forest plot for the impact of hyperphosphatemia on mortality. CI, confident interval; RR, relative risk.

Seven studies (Jung et al., 2016; Al Meshari et al., 2019; Jang et al., 2020; Miller et al., 2020; Wang et al., 2021; Li, Shen & Han, 2022; Xu et al., 2023) reported mortality in patients with high serum phosphate levels compared to those with normal serum phosphate levels (Fig. 2). There were 2,089 deaths in 6,041 patients with high serum phosphate levels (34.6%) and 3,224 deaths in 16,734 patients with normal serum phosphate levels (19.3%). Patients with high phosphate levels had a higher risk of death than those with normal serum phosphate levels (RR = 1.46; 95% CI [1.22–1.74]; *P* = 0.000). Heterogeneity analysis showed I^2^ = 87.3% and *P* = 0.000. Therefore, the random effects model (REM) was adopted.

In order to explore potential sources of heterogeneity, a subgroup analysis was performed according to whether the data outcomes were derived from single or multiple factors, and it was found that high serum phosphate levels were associated with a higher mortality risk across all groups. The single factor subgroup analysis results were (RR = 1.58; 95% CI [1.16–2.16]; *P* = 0.003), multiple factor subgroup analysis results were (RR = 1.35; 95% CI [1.19–1.54]; *P* = 0.000). More details are shown in Table 2.

**Table 2:** Results of meta-analysis.

Outcome	References	I^2^, *P*	RR, 95% CI	*P*	Begg’s, P	Egger’s, P
All-cause mortality						
Hyperphosphatemia *vs* Normophosphatemia						
Overall	Jung et al. (2016), Al Meshari et al. (2019), Jang et al. (2020), Miller et al. (2020), Wang et al. (2021), Li, Shen & Han (2022), Xu et al. (2023)	87.3%, 0.000	1.46 [1.22–1.74]	0.000	0.548	0.199
Subgroup analysis						
Univariable	Jung et al. (2016), Miller et al. (2020), Li, Shen & Han (2022)		1.58 [1.16–2.16]	0.003		
multivariable	Al Meshari et al. (2019), Jang et al. (2020), Wang et al. (2021), Xu et al. (2023)		1.35 [1.19–1.54]	0.000		
Hypophosphatemia *vs* Normophosphatemia						
Overall	Jung et al. (2016), Al Meshari et al. (2019), Jang et al. (2020), Miller et al. (2020), Wang et al. (2021), Xu et al. (2023)	0.0%, 0.698	0.97 [0.86–1.09]	0.588	1.000	0.239
Subgroup analysis						
Univariable	Jung et al. (2016), Miller et al. (2020)		0.81 [0.73–0.90]	0.985		
multivariable	Al Meshari et al. (2019), Jang et al. (2020), Wang et al. (2021), Xu et al. (2023)		0.96 [0.85–1.09]	0.578		
Per-increased						
Overall	Jung et al. (2016), Wang et al. (2020), Cao et al. (2021), Guo et al. (2022), Li, Shen & Han (2022), Xu et al. (2023)	62.2%, 0.021	1.15 [1.10–1.20]	0.000	1.000	0.892
Outcome	References	I^2^, P	WMD, 95% CI		Begg’s, P	Egger’s, P
ICU LOS						
Hyperphosphatemia *vs* Normophosphatemia						
Overall	Jung et al. (2016), Al Meshari et al. (2019), Wang et al. (2021), Guo et al. (2022), Li, Shen & Han (2022), Xu et al. (2023)	95.4%, 0.000	0.63 [0.27–0.98]	0.001	1.000	0.781
Hypophosphatemia *vs* Normophosphatemia						
Overall	Jung et al. (2016), Al Meshari et al. (2019), Wang et al. (2021), Guo et al. (2022), Li, Shen & Han (2022), Xu et al. (2023)	98.2%, 0.000	−0.23 [−0.75–0.29]	0.394	1.000	0.921
In-hospital LOS						
Hyperphosphatemia *vs* Normophosphatemia						
Overall	Jung et al. (2016), Al Meshari et al. (2019), Wang et al. (2021), Guo et al. (2022), Li, Shen & Han (2022), Xu et al. (2023)	97.7%, 0.000	0.22 [−0.61–1.05]	0.609	0.452	0.725
Hypophosphatemia *vs* Normophosphatemia						
Overall	Jung et al. (2016), Al Meshari et al. (2019), Wang et al. (2021), Guo et al. (2022), Li, Shen & Han (2022), Xu et al. (2023)	98.8%, 0.000	−0.62 [−1.72–0.49]	0.274	1.000	0.821

**Note:**

RR, relative risk; LOS, length of stay; WMD, weighted mean difference.

##### Hypophosphatemia vs Normophosphatemia

**Figure 3:**
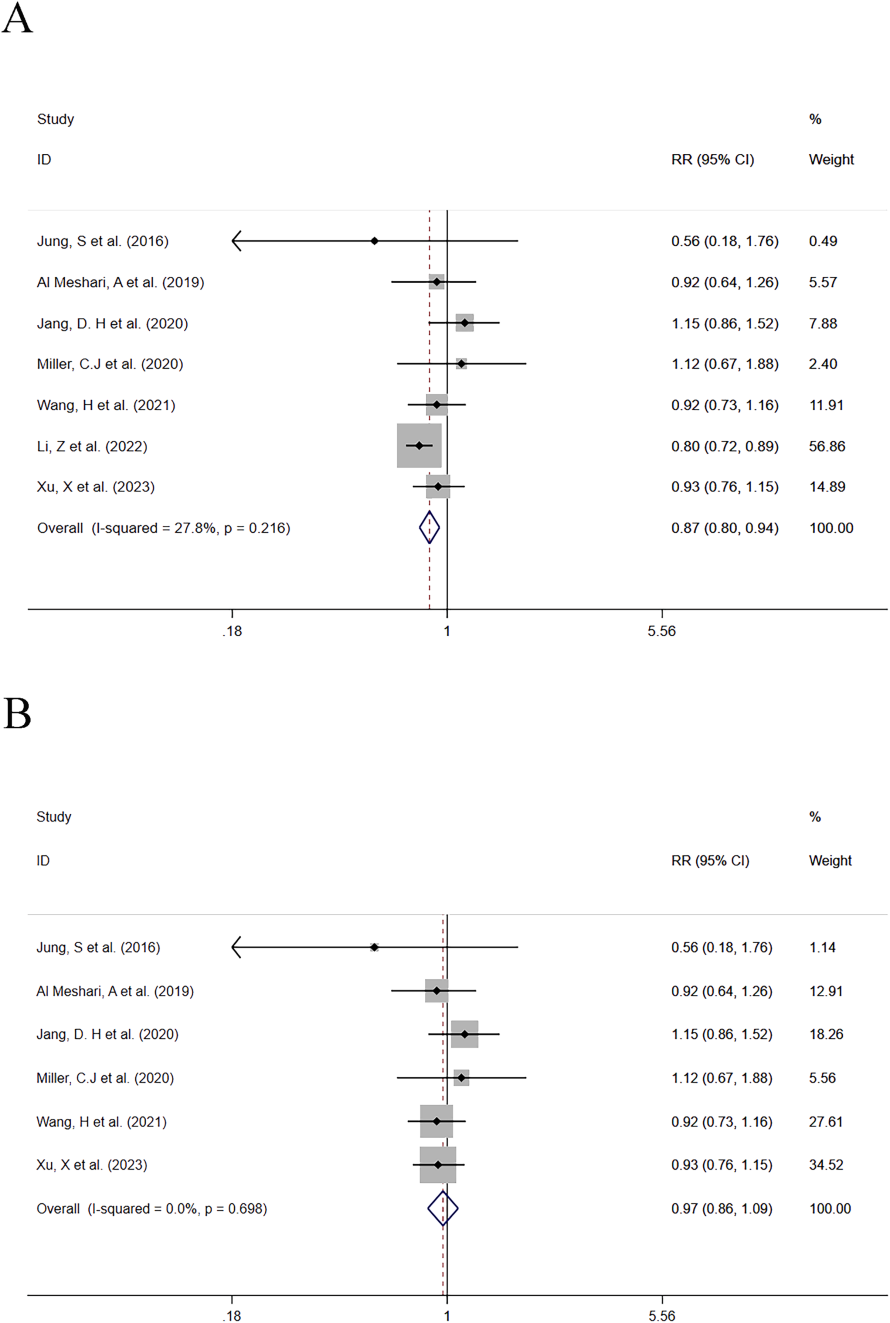
(A and B) Forest plot for the impact of hypophosphatemia on mortality. CI, confident interval; RR, relative risk.

In this group, seven studies compared the mortality between patients with low serum phosphate levels and normal serum phosphate levels. A total of 4,828 patients with low serum phosphate levels were involved including 725 deaths (15%). One study with a significant impact on the effect size of the outcome was eliminated from the combined analysis (Xu et al., 2023). Before the study was eliminated, the data combination results were (RR = 0.216; 95% CI [0.80–0.94]; *P* = 0.001) (Fig. 3A). It was found that low serum phosphate levels were associated with a reduced mortality risk, and the results were statistically significant. After the study was eliminated, the data were re-combined, and the results were (RR = 0.97; 95% CI [0.86–1.09]; *P* = 0.588) (Fig. 3B). It was found that low serum phosphate levels were associated with a reduced mortality risk, but the results were not statistically significant. Heterogeneity analysis showed that I^2^ was 0.0% and the *P*-value was 0.698. Therefore, the FEM was adopted. A subgroup analysis was made based on whether the data analysis of outcomes of each study was based on a single factor or multiple factors respectively. The detailed subgroup analysis results are provided in Table 2.

##### Per-increased

**Figure 4:**
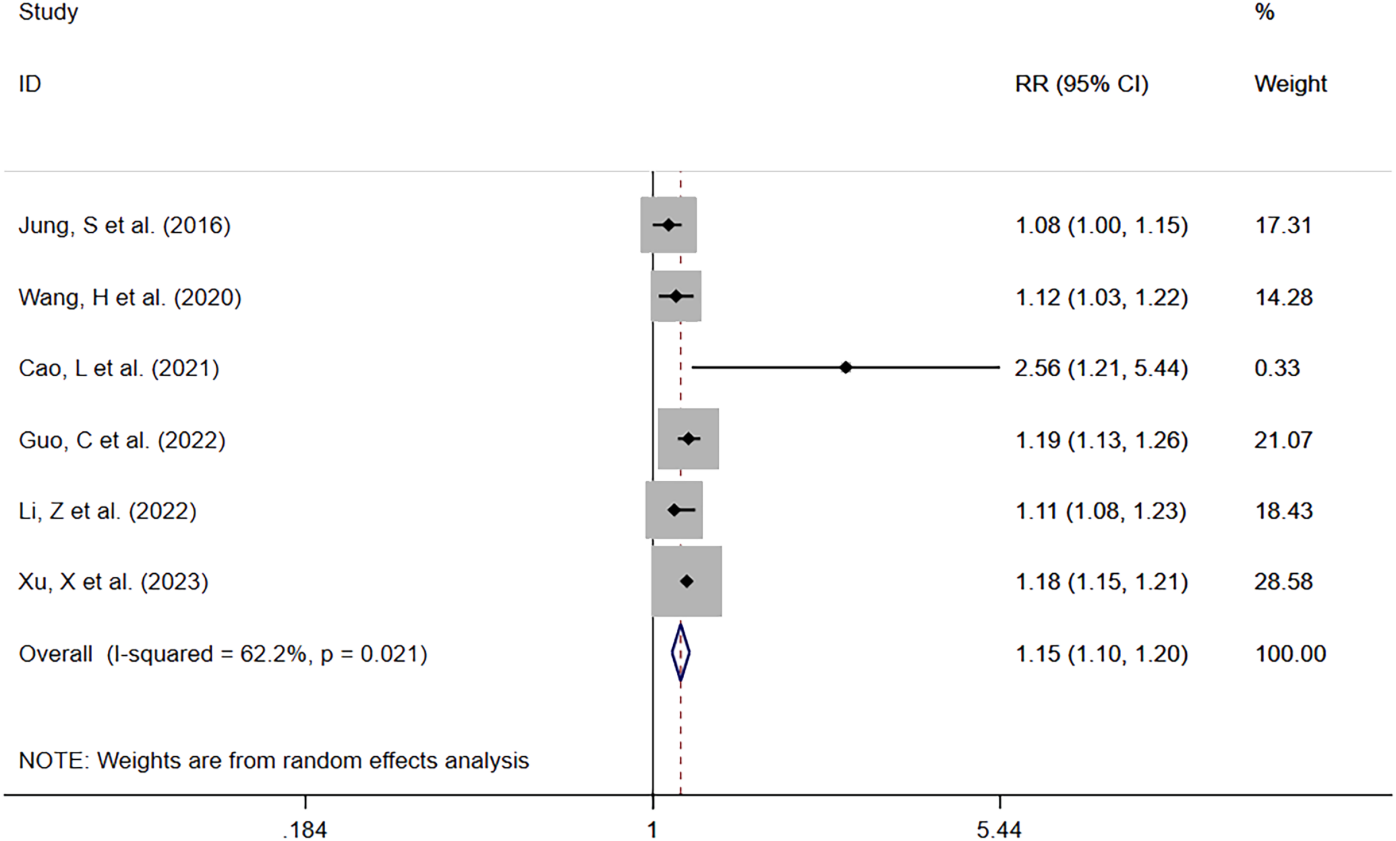
Forest plot for the impact of per-increased in serum phosphate on mortality. CI, confident interval; RR, relative risk.

Six studies reported serum phosphate levels as a continuous variable (Jung et al., 2016; Wang et al., 2020; Cao et al., 2021; Guo et al., 2022; Li, Shen & Han, 2022; Xu et al., 2023). Except for the study by Wang et al. (2020), which did not provide the number of deaths, a total of 24,724 patients with 6,222 deaths (25.2%) were involved in the other five studies. It was found that increased serum phosphate levels were associated with mortality risk. The meta-analysis showed that the mortality risk was elevated with increasing serum phosphate levels (RR = 1.15; 95% CI [1.10–1.20]; *P* = 0.000) (Fig. 4). Heterogeneity analysis showed I^2^ = 62.2% and *P* = 0.021. Therefore, the REM was adopted.

### Secondary outcome

#### ICU LOS

##### Hyperphosphatemia vs Normophosphatemia

**Figure 5:**
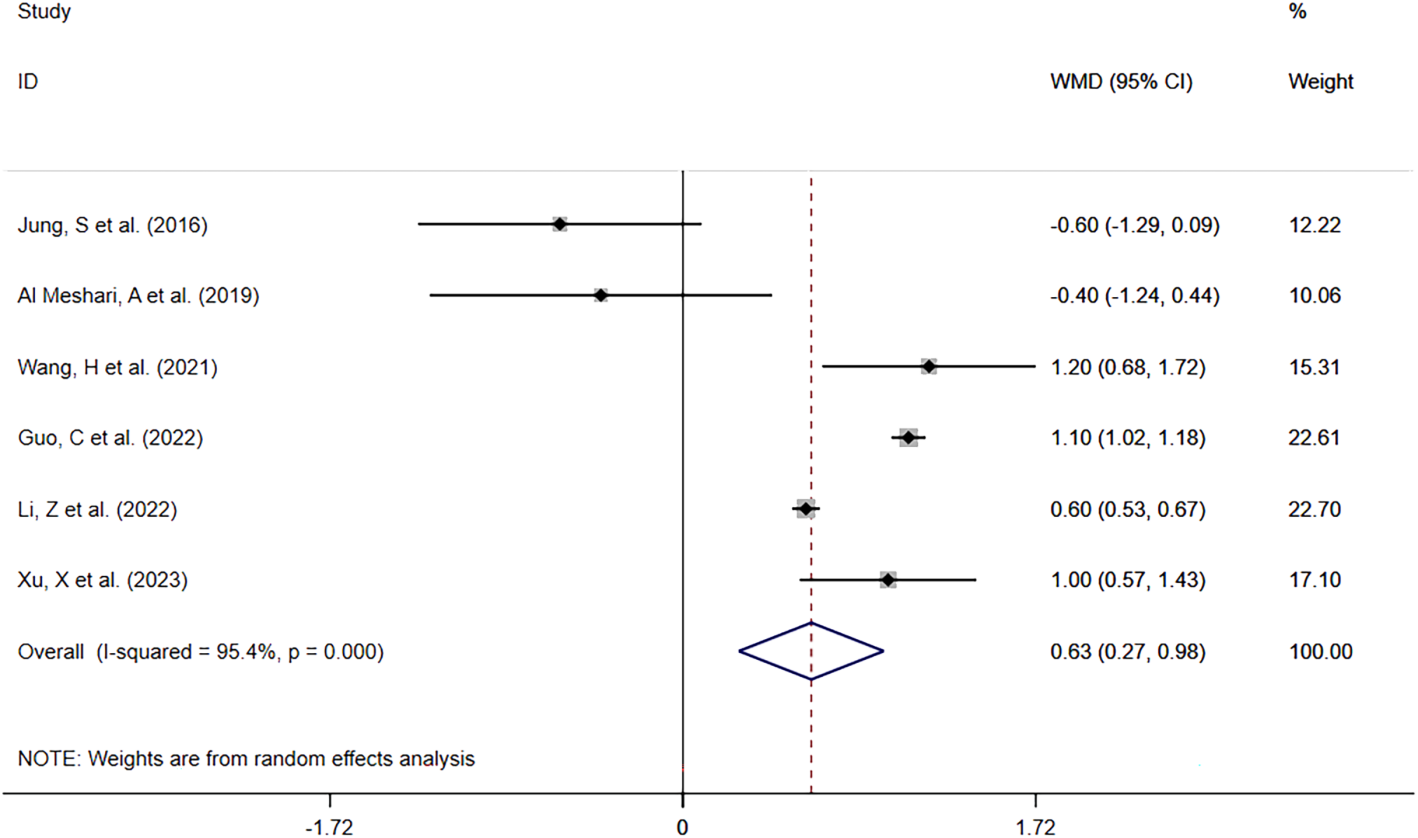
Forest plot for the impact of hyperphosphatemia on ICU LOS. CI, confident interval; WMD, weighted mean difference.

Six included studies (Jung et al., 2016; Al Meshari et al., 2019; Wang et al., 2021; Guo et al., 2022; Li, Shen & Han, 2022; Xu et al., 2023) reported ICU LOS in 6,764 patients with high serum phosphate levels against 17,986 with normal serum phosphate levels. The meta-analysis (Fig. 5) showed that high phosphate levels were significantly associated with prolonged ICU LOS (WMD = 0.63; 95% CI [0.27–0.98]; *P* = 0.001). Heterogeneity analysis showed I^2^ = 95.4% and *P* = 0.000. Therefore, the REM was adopted.

##### Hypophosphatemia vs Normophosphatemia

**Figure 6:**
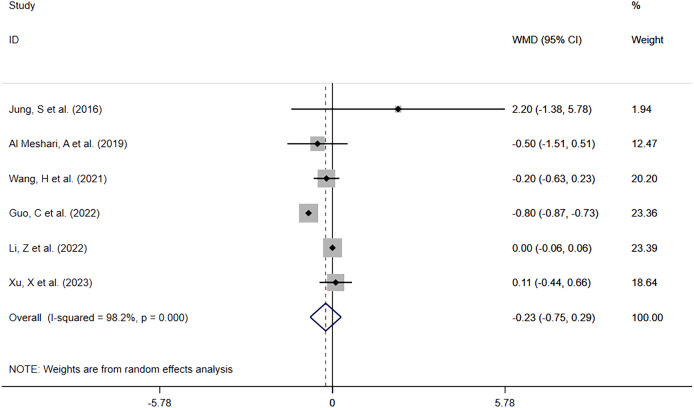
Forest plot for the impact of hypophosphatemia on ICU LOS. CI, confident interval; WMD, weighted mean difference.

Of the same six studies, 5,869 patients with low serum phosphate levels were involved in the comparison of ICU LOS between the low serum phosphate level group and the normal serum phosphate level group. The meta-analysis (Fig. 6) showed that hypophosphatemia was not associated with prolonged ICU LOS (WMD= −0.23; 95% CI [−0.75–0.29]; *P* = 0.394). Heterogeneity analysis showed I^2^ = 98.2% and *P* = 0.000. Therefore, the REM was adopted.

#### In-hospital LOS

##### Hyperphosphatemia vs Normophosphatemia

**Figure 7:**
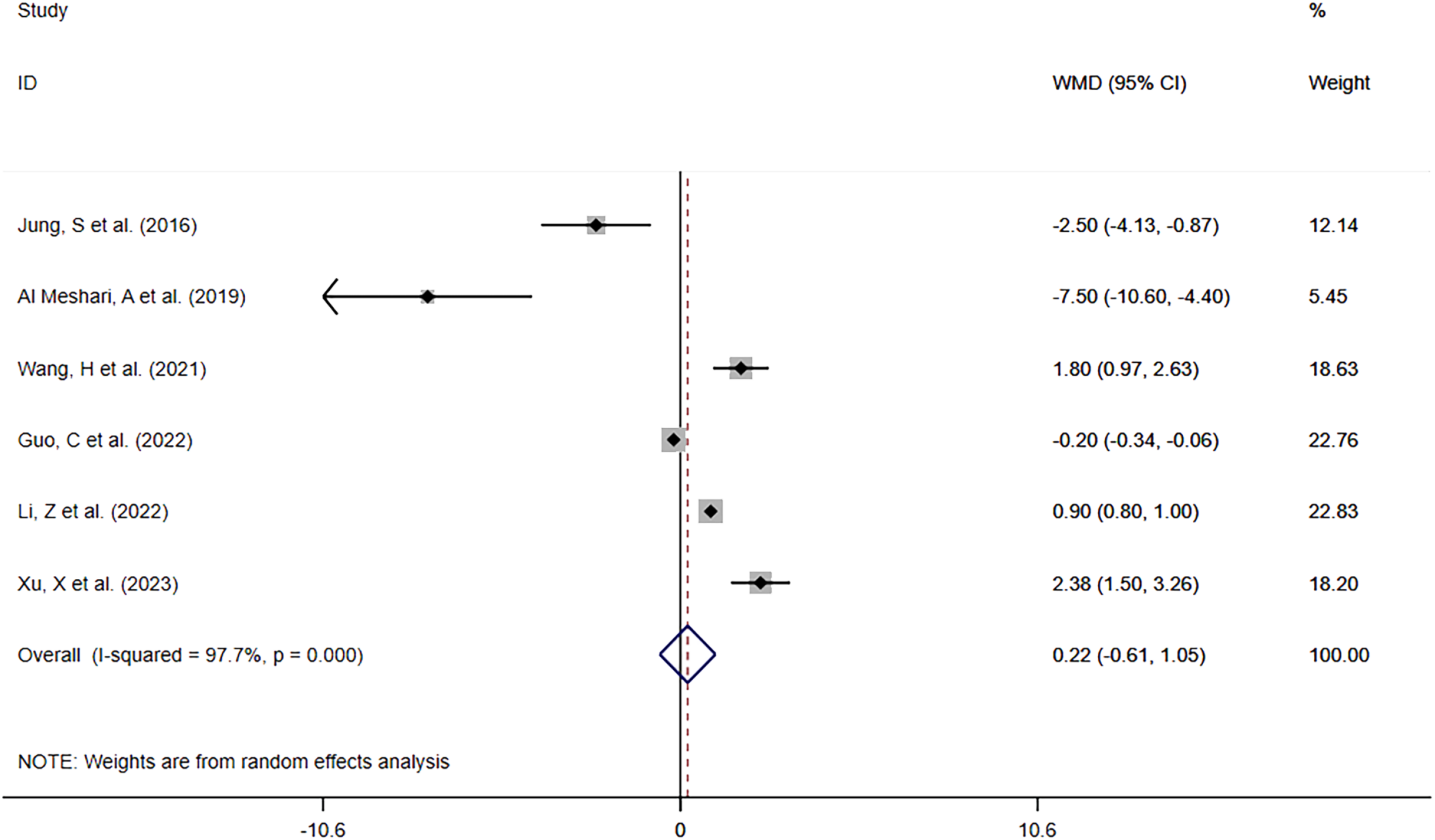
Forest plot for the impact of hyperphosphatemia on In-hospital LOS. CI, confident interval; WMD, weighted mean difference.

Six studies (Jung et al., 2016; Al Meshari et al., 2019; Wang et al., 2021; Guo et al., 2022; Li, Shen & Han, 2022; Xu et al., 2023) reported in-hospital LOS in patients with high phosphate levels compared to those with normal phosphate levels. The meta-analysis (Fig. 7) showed that hyperphosphatemia before interventional treatment was somewhat associated with prolonged in-hospital LOS (WMD = 0.22; 95% CI [−0.61–1.05]; *P* = 0.609), without significant difference. Heterogeneity analysis showed I2 = 97.7% and *P* = 0.000. Therefore, the REM was adopted.

##### Hypophosphatemia vs Normophosphatemia

**Figure 8:**
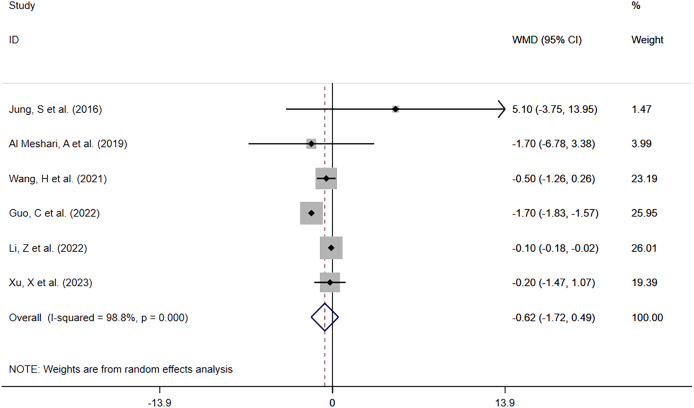
Forest plot in assessing the impact of hypophosphatemia on In-hospital LOS. CI, confident interval; WMD, weighted mean difference.

The same six studies reported in-hospital LOS between patients with low phosphate levels and patients with normal phosphate levels. The meta-analysis (Fig. 8) showed that hypophosphatemia before interventional treatment was not associated with prolonged in-hospital LOS (WMD = −0.62; 95% CI [−1.72–0.49]; *P* = 0.274). Heterogeneity analysis showed that I^2^ was 98.8% and the *P*-value was 0.000. Therefore, the REM was adopted.

### Sensitivity analysis and publication bias

A sensitivity analysis was performed for all the outcomes above separately to investigate the stability of the outcomes by eliminating the literature one by one. In terms of the outcome of all-cause mortality between patients with low serum phosphate levels and normal serum phosphate levels, it was found that the study by Li, Shen & Han (2022) had a significant impact on the meta-analysis results, and this article was eliminated from the analysis. The sensitivity analysis results for other outcomes were relatively robust. Publication bias assessment was performed on all outcomes. For the outcome of all-cause mortality, the *P*-value in Begg’s test and Egger’s test was 0.548 and 0.199 (hyperphosphatemia *vs* normal phosphate level group), 1.000 and 0.239 (hypophosphatemia *vs* normal phosphate level group), and 1.000 and 0.892 (Per-increased group) respectively. For ICU LOS, the *P*-value in Begg’s test and Egger’s test was 1.000 and 0.781 (hyperphosphatemia *vs* normal phosphate level group), and 1.000 and 0.921 (hypophosphatemia *vs* normal phosphate level group) respectively. In terms of in-hospital LOS, the *P*-value in Begg’s test and Egger’s test was 0.452 and 0.725 (hyperphosphatemia *vs* normal phosphate level group), and 1.000 and 0.821 (hypophosphatemia *vs* normal phosphate level group) respectively. These results and relevant tables and figures are shown in Table 2 and File S1.

## Discussion

This meta-analysis included ten studies involving nearly 40,000 patients with different serum phosphate levels. The current study is the first-ever systematic review and meta-analysis to investigate the impact of different phosphate levels on clinical prognosis outcomes of sepsis. Our final study evidence showed that patients with hyperphosphatemia before intervention were significantly associated with an increased risk of all-cause mortality and prolonged ICU LOS compared to patients with normal serum phosphate levels, while patients with hypophosphatemia before intervention were not significantly associated with an increased risk of mortality and prolonged LOS.

The meta-analysis results from the hyperphosphatemia *vs* normal phosphate level group showed that sepsis patients with high serum phosphate levels before intervention were positively correlated with an increased risk of all-cause mortality and prolonged ICU hospitalization, and possibly associated with prolonged in-hospital LOS with no statistically significant difference. In order to further verify this observation result, the studies using mortality of sepsis patients with the serum phosphate level as the continuous variable were included in the analysis of all-cause mortality, and it was found that elevated serum phosphate levels were associated with a higher mortality risk in sepsis patients. The findings were consistent with the results reported in a previous meta-analysis study on the relationship between high serum phosphate levels and all-cause mortality and ICU LOS in critically ill patients (Zheng et al., 2022). However, the subjects of the previous review are critically ill patients, with a wide spectrum of disease types and specialties. In addition, there is a lack of discussion on the causes of elevated phosphate levels among different disease types, and there was a possible bias in the relationship between high serum phosphate levels and mortality due to different etiologies. In contrast, the subjects of our study are a single patient population with sepsis, which reduces the probability of bias in the prognostic outcome of high serum phosphate levels among multiple disease types and multiple factors to some extent. In addition, the studies not included in the previous review with the serum phosphate level as a continuous variable were also included in our study, which enriched our study, and the sample size was large enough to perform statistical analysis, thus providing more robust meta-analysis results.

In our study, the mortality of sepsis patients with high serum phosphate levels was 34.6%. On the one hand, the high mortality may be associated with the fact that the sepsis patients enrolled had a mean age above 60 at baseline and were complicated with a previous history of long-term chronic diseases, because older patients were more closely associated with a high incidence of cardiovascular diseases and significantly decrease in somatic function (Wannamethee, 2022), with poor prognosis. However, this finding needs to be verified in young sepsis populations not complicated with long-term chronic diseases. On the other hand, high serum phosphate levels result in an increased risk of poor prognosis such as mortality in septic patients, primarily because of the underlying mechanism that phosphate exists in human cells primarily in the form of organophosphate compounds, including adenosine triphosphate (ATP, London, UK), creatine phosphate, and adenosine monophosphate, which provide energy for tissue through cellular mitochondrial respiratory chains and oxidative phosphorylation. Therefore, the dyshomeostasis of phosphate reduces the energy source for the tissue and increases energy consumption, causing vascular endothelial cell dysfunction and the release of phosphate-induced mitochondrial active oxygen, increased mortality risk, and prolonged LOS. In addition, many studies have reported that phosphate is also closely associated with coronary vascular calcification and secondary hyperparathyroidism in the human body and is itself a risk factor for the onset of cardiovascular diseases and all-cause mortality. It mainly damages cardiovascular function through multiple mechanisms such as inflammation, oxidative stress and induced cytotoxicity, while cardiovascular dysfunction is an important pathological process of sepsis. This suggests that sepsis patients at an increased risk of poor prognosis may be more sensitive to elevated phosphate levels (Ganesh et al., 2001; Smolenski et al., 1990; Block et al., 2004; Floege et al., 2011; Hruska et al., 2008; Oliveira & Kowaltowski, 2004). In addition to excessive intake of phosphate and reduced excretion, elevated phosphate levels are mainly attributable to tissue ischemic injury and cell membrane damage in sepsis patients, which leads to the release of intracellular phosphate into the blood and high serum phosphate levels. It has been previously reported that phosphate and lactate levels are elevated with prolonged ischemic injury in a similar pattern, and the phosphate concentration is positively correlated with the lactate concentration (McGovern et al., 2013; Tentori et al., 2008).

Our study showed that low phosphate levels were not associated with sepsis mortality and were possibly associated with shortened ICU LOS and in-hospital LOS, but the results were not statistically significant. However, previous studies have reported that low phosphate levels are associated with the prognosis of sepsis patients (Miller et al., 2020; Xu et al., 2023). Differences in prognostic outcomes of sepsis patients with low phosphate levels may be due to different cut-off points of low phosphate levels and testing time points used in different studies. For example, Xu et al. (2023) used phosphate test values of sepsis patients on the second day after admission to ICU, and found that low phosphate levels were a protective factor for the prognosis of sepsis patients and were independently associated with better prognosis. Moreover, different conclusions may be drawn on the relationship between low phosphate levels and the prognosis of sepsis due to the presence of confounding correction factors and the difference in their contents. Unlike the outcome of high serum phosphate levels, low phosphate levels did not result in higher mortality, probably because organ dysfunction and ischemic tissue damage due to clinical sepsis were more severe than cell function change and hypoxia caused by hypophosphatemia.

Several important limitations need to be considered in this meta-analysis. First of all, this review was limited by the inclusion of retrospective observational cohort studies, in which there may be unmeasurable confounding factors and recalling bias (Sedgwick, 2012). However, these confounding factors may not be addressed by robust study-specific adjustment and meta-analysis methods. Secondly, it’s a pity that a subgroup analysis of different continents or ethnic populations cannot be performed because almost all the populations enrolled in the study were from Asia. Finally, a subgroup analysis of all-cause mortality was made only based on single-factor and multiple-factor analysis. However, the source of heterogeneity cannot be interpreted according to the findings. The number of outcomes included in individual studies of studies was small, which may cause bias in the results. For example, in the study conducted by Jung et al. (2016) there were only 6 patients with hypophosphatemia including two deaths.

## Conclusion

In conclusion, compared to sepsis patients with normal serum phosphate levels, sepsis patients with high serum phosphate levels had a significantly increased risk of all-cause mortality and prolonged ICU LOS, but there was no significant difference in in-hospital LOS. Sepsis patients with low serum phosphate levels were not associated with the risk of all-cause mortality, ICU LOS, and in-hospital LOS. These findings support early control of serum phosphate levels in patients diagnosed with sepsis complicated with hyperphosphatemia. Due to the small number of included studies and the large proportion of retrospective cohort studies, more high-quality, multicenter, large-sample prospective studies are warranted in the future.

## Supplemental Information

10.7717/peerj.16241/supp-1 Supplemental Information 1 Suppplementary tables and figures.

10.7717/peerj.16241/supp-2 Supplemental Information 2 PRISMA checklist.

10.7717/peerj.16241/supp-3 Supplemental Information 3 Systematic Review and/or Meta-Analysis Rationale.

## Additional Information and Declarations

### Data Availability

The following information was supplied regarding data availability: This is a systematic review.
