# Supplementary material for: Prognostic value of serum phosphate levels in sepsis: a systematic review and meta-analysis
Source: PeerJ. 2023 Oct 13;11:e16241. doi: 10.7717/peerj.16241 (PMC10578301; doi:10.7717/peerj.16241)
Supplement: Supplemental Information 1 [file peerj-11-16241-s001.docx]

**Supplementary File 1**

**1.Searching strategies**

**Pubmed**

| 1 | "Sepsis"[Mesh] |
| --- | --- |
| 2 | "Shock, Septic"[Mesh] |
| 3 | ("Shock, Septic"[Mesh]) OR ("Sepsis"[Mesh]) |
| 4 | (((((((Sepsis) OR (septic)) OR (Pyemia*)) OR (Pyohemia*)) OR (Pyaemia*)) OR (Septicemia*)) OR (Septic shock)) OR (Septic) |
| 5 | ((((((((Sepsis) OR (septic)) OR (Pyemia*)) OR (Pyohemia*)) OR (Pyaemia*)) OR (Septicemia*)) OR (Septic shock)) OR (Septic)) OR (("Shock, Septic"[Mesh]) OR ("Sepsis"[Mesh])) |
| 6 | (Mortality[MeSH Terms]) OR (Death[MeSH Terms]) |
| 7 | ((Mortalit*) OR (Fatalit*)) OR (Death*) |
| 8 | ((Mortality[MeSH Terms]) OR (Death[MeSH Terms])) OR (((Mortalit*) OR (Fatalit*)) OR (Death*)) |
| 9 | ((Phosphate[MeSH Terms]) OR (Hyperphosphatemia[MeSH Terms])) OR (Hypophosphatemia[MeSH Terms]) |
| 10 | (((Phosphate*) OR (Orthophosphate*)) OR (Hyperphosphatemia*)) OR (Hypophosphatemia*) |
| 11 | (((Phosphate[MeSH Terms]) OR (Hyperphosphatemia[MeSH Terms])) OR (Hypophosphatemia[MeSH Terms])) OR ((((Phosphate*) OR (Orthophosphate*)) OR (Hyperphosphatemia*)) OR (Hypophosphatemia*)) |
| 12 | (((((Phosphate[MeSH Terms]) OR (Hyperphosphatemia[MeSH Terms])) OR (Hypophosphatemia[MeSH Terms])) OR ((((Phosphate*) OR (Orthophosphate*)) OR (Hyperphosphatemia*)) OR (Hypophosphatemia*))) AND (((Mortality[MeSH Terms]) OR (Death[MeSH Terms])) OR (((Mortalit*) OR (Fatalit*)) OR (Death*)))) AND (((((((((Sepsis) OR (septic)) OR (Pyemia*)) OR (Pyohemia*)) OR (Pyaemia*)) OR (Septicemia*)) OR (Septic shock)) OR (Septic)) OR (("Shock, Septic"[Mesh]) OR ("Sepsis"[Mesh]))) |

**Cochrane Library**

| #1 | MeSH descriptor: [Sepsis] explode all trees |
| --- | --- |
| #2 | MeSH descriptor: [] explode all trees |
| #3 | (Sepsis or Pyemia* or Pyohemia* or Pyaemia* or Septicemia* or 'Septic shock' or Septic) |
| #4 | MeSH descriptor: [Mortality] explode all trees |
| #5 | MeSH descriptor: [Death] explode all trees |
| #6 | (Mortalit* or Fatalit* or Death*) |
| #7 | MeSH descriptor: [Phosphates] explode all trees |
| #8 | MeSH descriptor: [Hyperphosphatemia] explode all trees |
| #9 | MeSH descriptor: [Hypophosphatemia] explode all trees |
| #10 | (Phosphate* or Orthophosphate* or Hyperphosphatemia* or Hypophosphatemia*) |
| #11 | #1 or #2 or #3 |
| #12 | #4 or #5 or #6 |
| #13 | #7 or #8 or #9 or #10 |
| #14 | #11 and #12 and #13 |

**Web of Science**

'Mortalit*' or 'Fatalit*' or 'Death*'  (Topic)  and Sepsis or Pyemia* or Pyohemia* or Pyaemia* or Septicemia* or 'Septic shock' or Septic (Topic)  and Phosphate* or Orthophosphate* or Hyperphosphatemia* or Hypophosphatemia*  (Topic) 377

**Embase**

| #1 | 'sepsis'/exp OR sepsis OR pyemia* OR pyohemia* OR pyaemia* OR septicemia* OR 'septic shock' OR septic |
| --- | --- |
| #2 | 'mortality'/exp OR 'death'/exp OR mortalit* OR fatalit* OR death* |
| #3 | 'phosphate'/exp OR 'hyperphosphatemia'/exp OR 'hypophosphatemia'/exp OR phosphate* OR orthophosphate* OR hyperphosphatemia* OR hypophosphatemia* |
| #4 | #1 AND #2 AND #3 |

**2.Quality assessment**

| **Study** | **Selection** | | | | **Comparability** | **Outcome** | | | **Quality scores** |
| --- | --- | --- | --- | --- | --- | --- | --- | --- | --- |
|  | Representativeness of the exposed cohort | Selection of the nonexposed cohort | Ascertainment of exposure | Demonstration that outcome of interest was not present at start of study | Comparability of cohorts on the basis of the design or analysis | Assessment of outcome | Was follow-up long enough for outcomes to occur | Adequacy of follow up of cohorts |  |
| Jung, S et al. 2016 | **⭐** | **⭐** | **⭐** | **⭐** | **⭐** | **⭐** | **⭐** | **⭐** | 8 |
| Al Meshari, A et al. 2019 | **⭐** | **⭐** | **⭐** | **⭐** | **⭐⭐** | **⭐** | **⭐** | **⭐** | 9 |
| Jang, D. H et al. 2020 | **⭐** | **⭐** | **⭐** | **⭐** | **⭐⭐** | **⭐** | **⭐** | **⭐** | 9 |
| Miller, C. J et al. 2020 | **⭐** | **⭐** | **⭐** | **⭐** | **⭐** | **⭐** | **⭐** | **⭐** | 8 |
| Wang, H et al. 2020 | **⭐** | **⭐** | **⭐** | **⭐** | **⭐⭐** | **⭐** | **⭐** | **⭐** | 9 |
| Cao, L et al. 2021 | **⭐** | **⭐** | **⭐** | **⭐** | **⭐⭐** | **⭐** | **⭐** | **⭐** | 9 |
| Wang, H et al. 2021 | **⭐** | **⭐** | **⭐** | **⭐** | **⭐⭐** | **⭐** | **⭐** | **⭐** | 9 |
| Guo, C et al. 2022 | **⭐** | **⭐** | **⭐** | **⭐** | **⭐⭐** | **⭐** | **⭐** | **⭐** | 9 |
| Li, Z et al. 2022 | **⭐** | **⭐** | **⭐** | **⭐** | **⭐** | **⭐** | **⭐** | **⭐** | 8 |
| Xu, X et al. 2023 | **⭐** | **⭐** | **⭐** | **⭐** | **⭐⭐** | **⭐** | **⭐** | **⭐** | 9 |

**Abbreviations:** H=high quality; M=moderate quality; L= low quality.

**Note:** A study was given a maximum of one point in each item within the patient selection and outcome domains and given a maximum of two points for the Comparability domain with the following criteria:

1. **Representation of the exposed cohort**：Studies received 1 point if they recruited consecutive series of adult patients with blood phosphate concentration tested, or all included patients or did not miss a large number of patients.

2. **Selection of the non-exposed cohort**：Studies received 1 point if both groups of patients （exposed and non-exposed）were recruited from the same cohort.

3. **Ascertainment of exposure**: Studies received 1 point if they had been demonstrated to have abnormal blood phosphate concentration.

4. **Outcome of interest was not present at start of study**: Studies received points if they demonstrated the outcome of interest was not present at the start of the study.

5. **Comparability:** Studies received points if they controlled the disease severity (i.e., SOFA, SAPS3, ISS or APACHEII scores) (1 point); or any additional important factors such as age, gender or ethnicities, comorbidities, or there were no significant differences between hyperphosphatemia/hypophosphatemia and normal phosphate concentration (1 point).

6. **Assessment of outcome**: Studies received 1 point if they had independent blind assessment or record linkage.

7. **Was follow-up long enough for outcomes to occur**: Studies received 1 point if they follow up until at least either inpatient mortality or for 30 days or had adequate record linkage.

8. **Adequacy of follow up for cohorts**: Studies received 1 point if all recruited subjects were all followed up, or the number lost to follow-up was unlikely to introduce bias (≤10%).

**3. Sensitivity analysis of Primary outcome.**

**3.1 *Hyperphosphatemia vs Normophosphatemia*, all-cause mortality.**


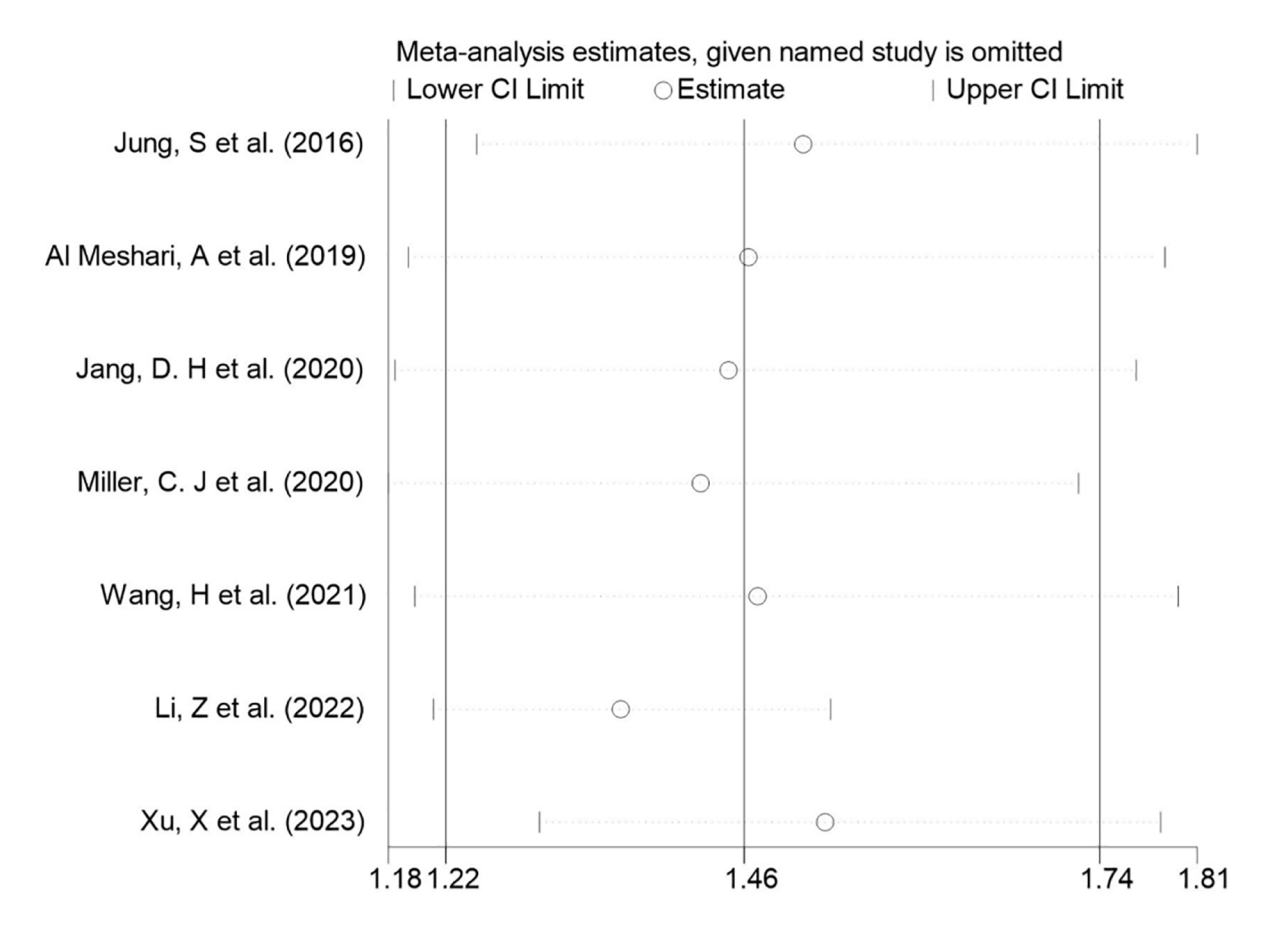


**3.2 *Hypophosphatemia vs Normophosphatemia, all-cause mortality.***


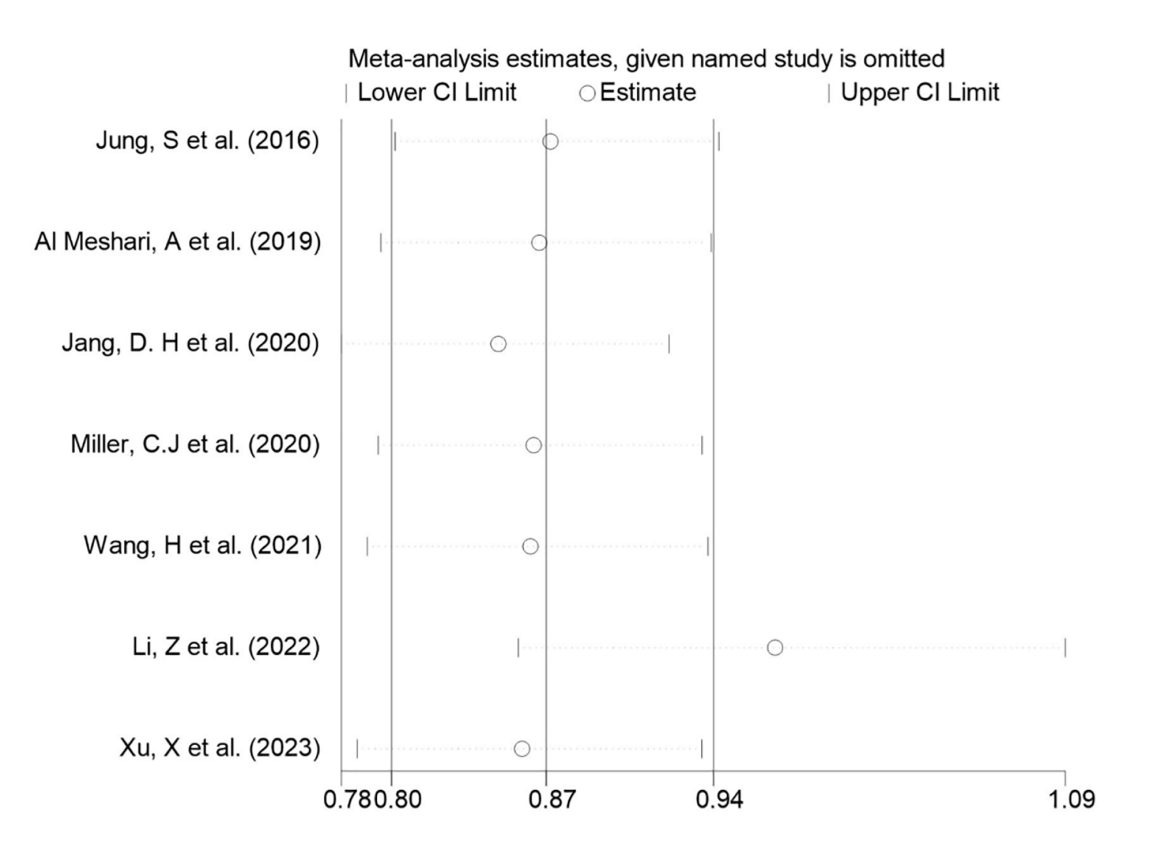


**3.3 *Per-increased***

**
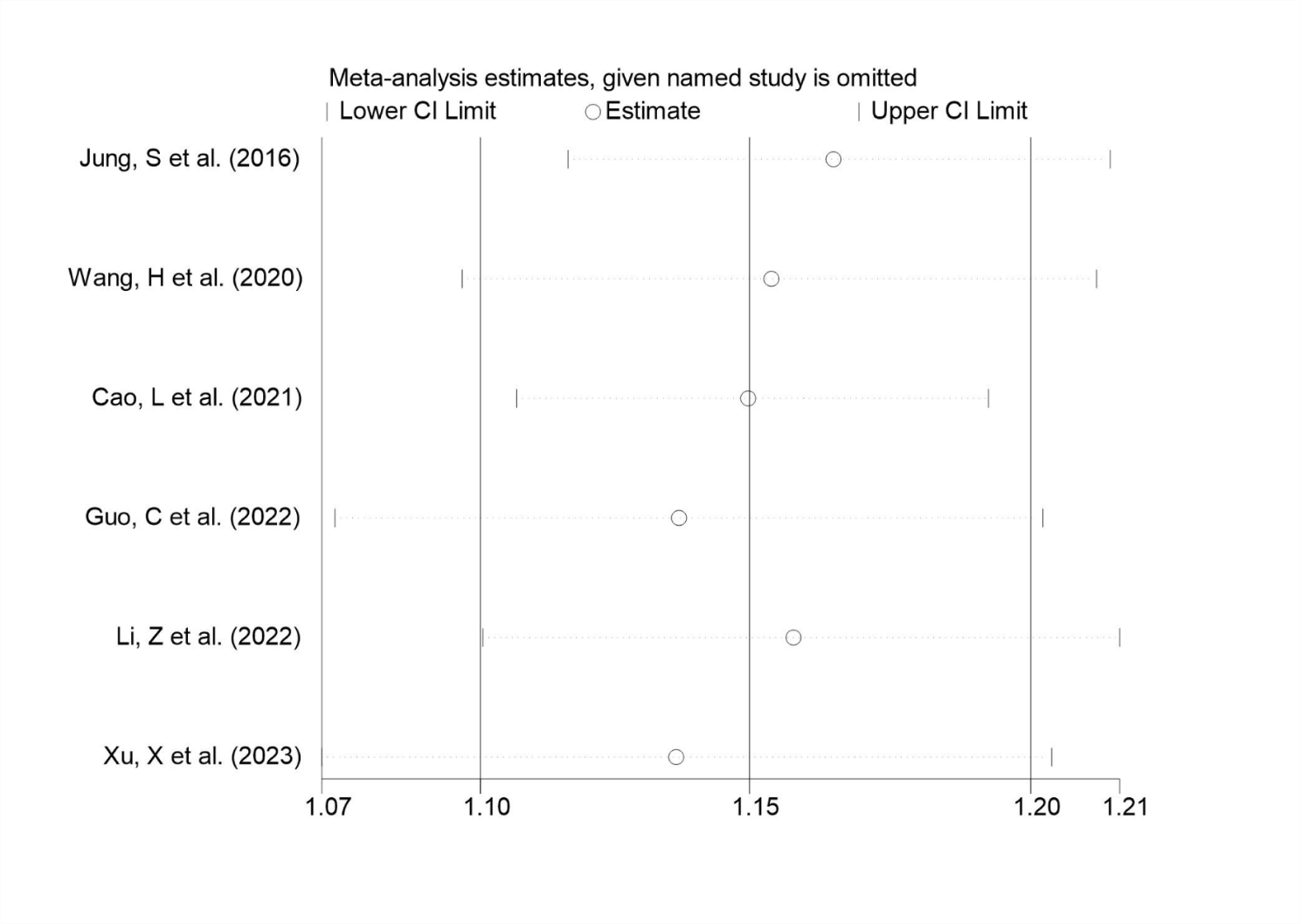
**

**4. *Sensitivity analysis of Secondary outcome.***

**4.1 *Hyperphosphatemia vs Normophosphatemia*.**

**ICU LOS**


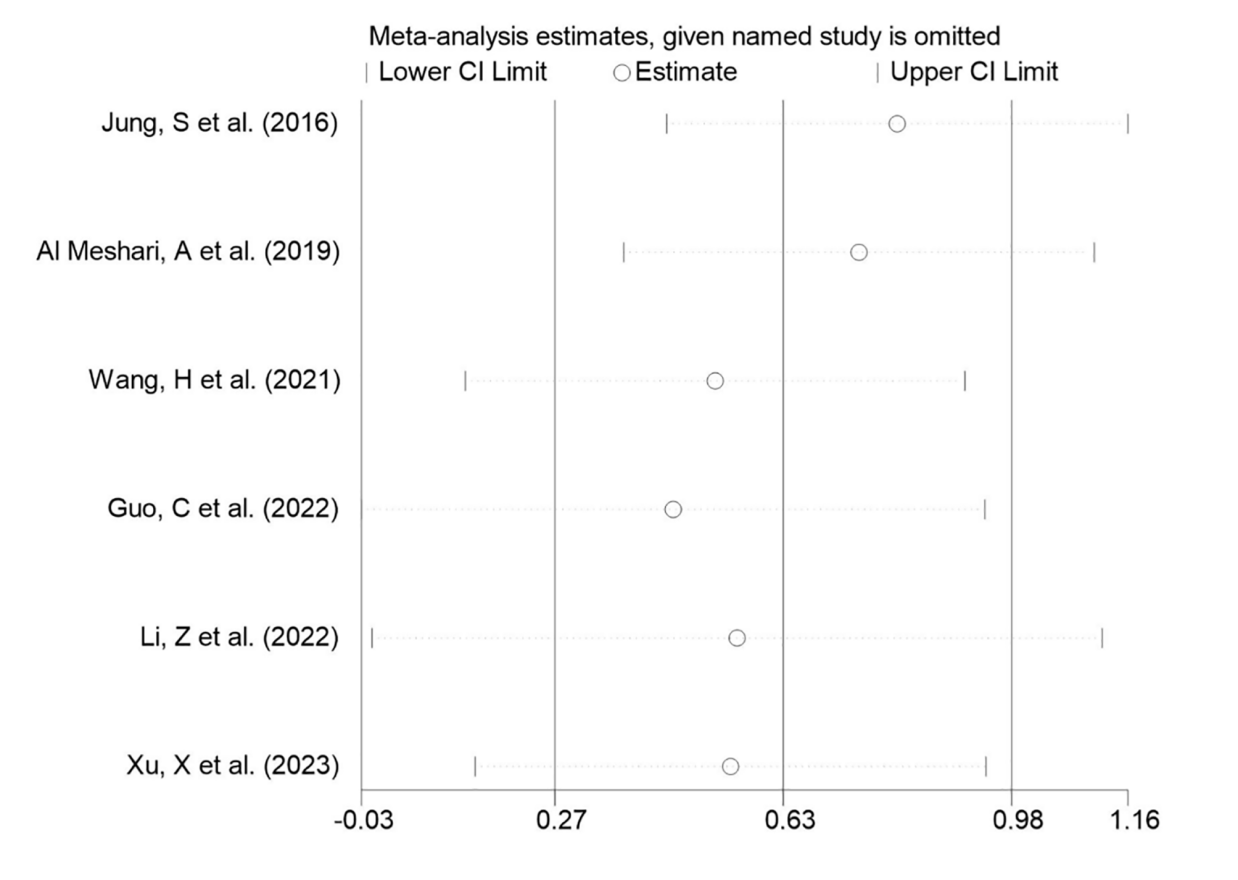


**In-hospital LOS**


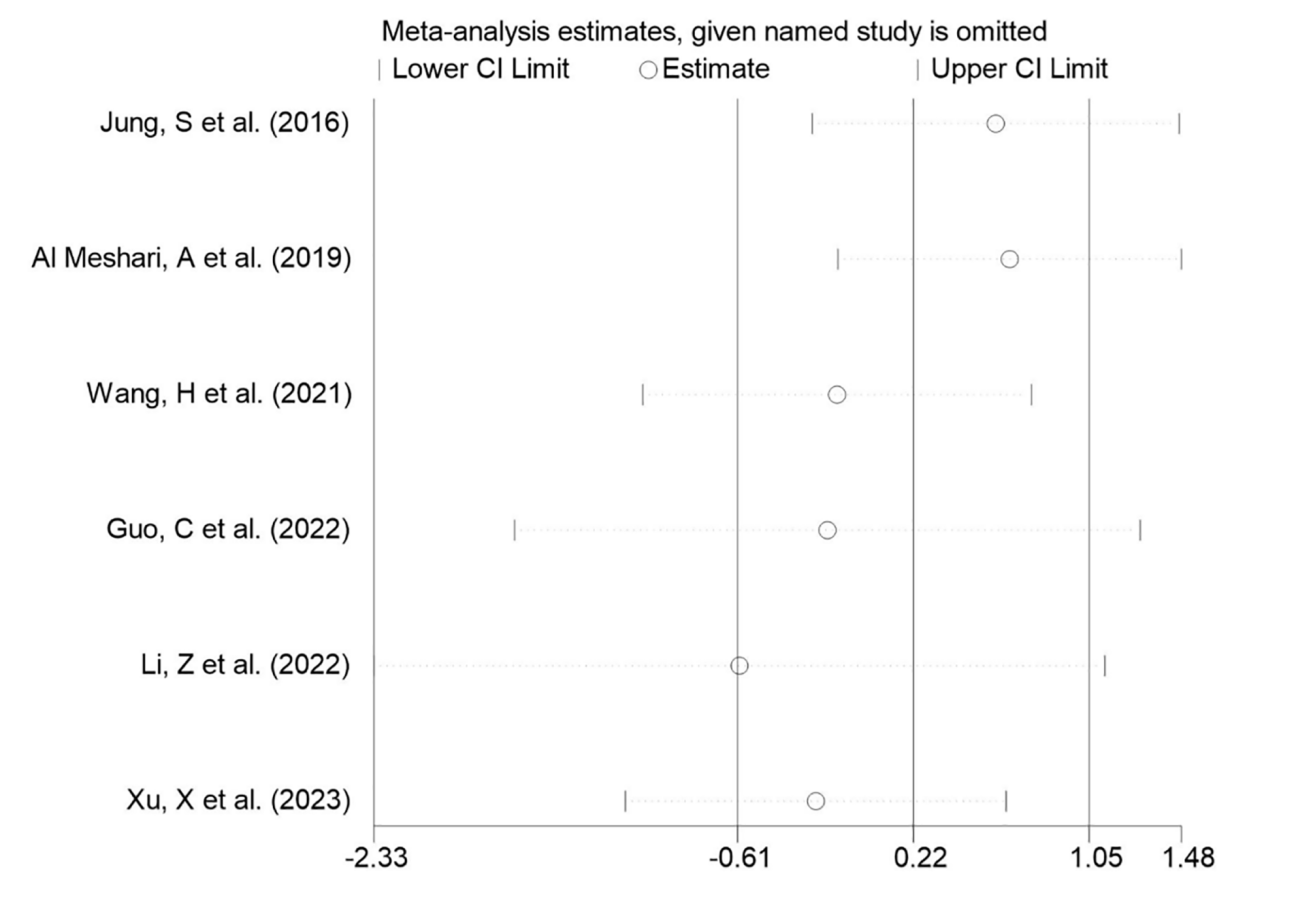


**4.2 *Hypophosphatemia vs Normophosphatemia.***

**ICU LOS**


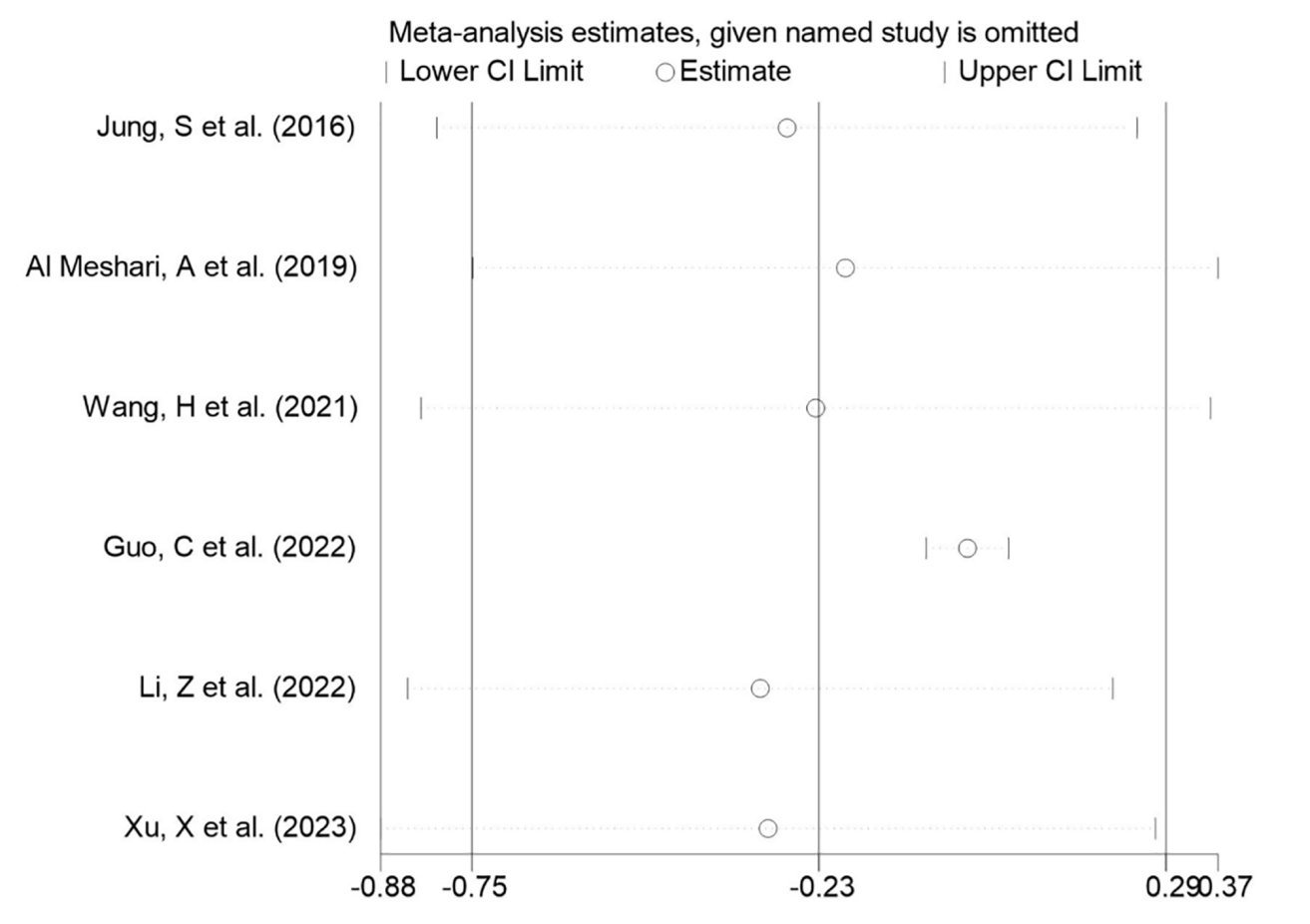


**In-hospital LOS**


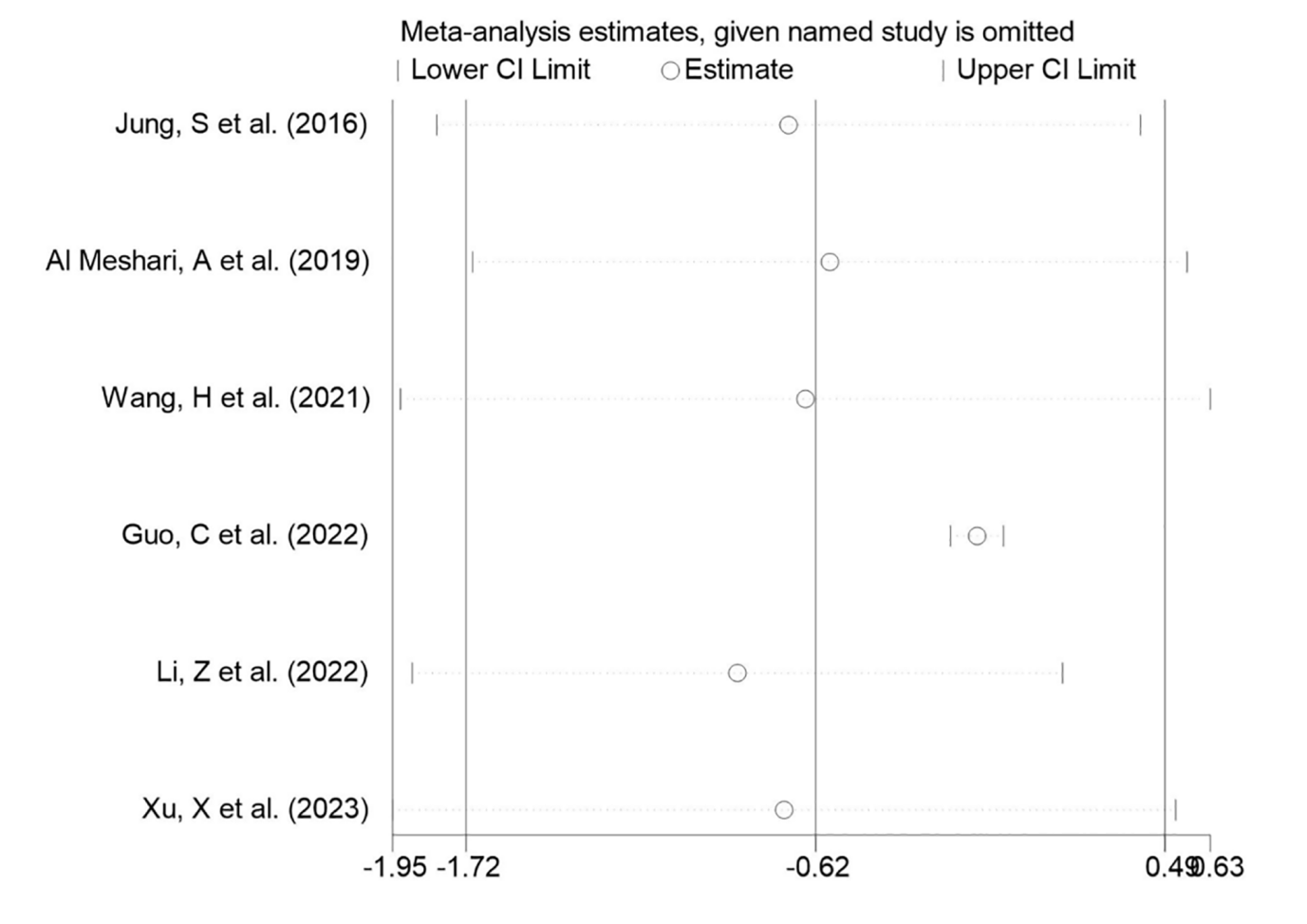


**5. Publication bias of Primary outcome.**

**5.1 *Hyperphosphatemia vs Normophosphatemia*. All-cause mortality.**

**
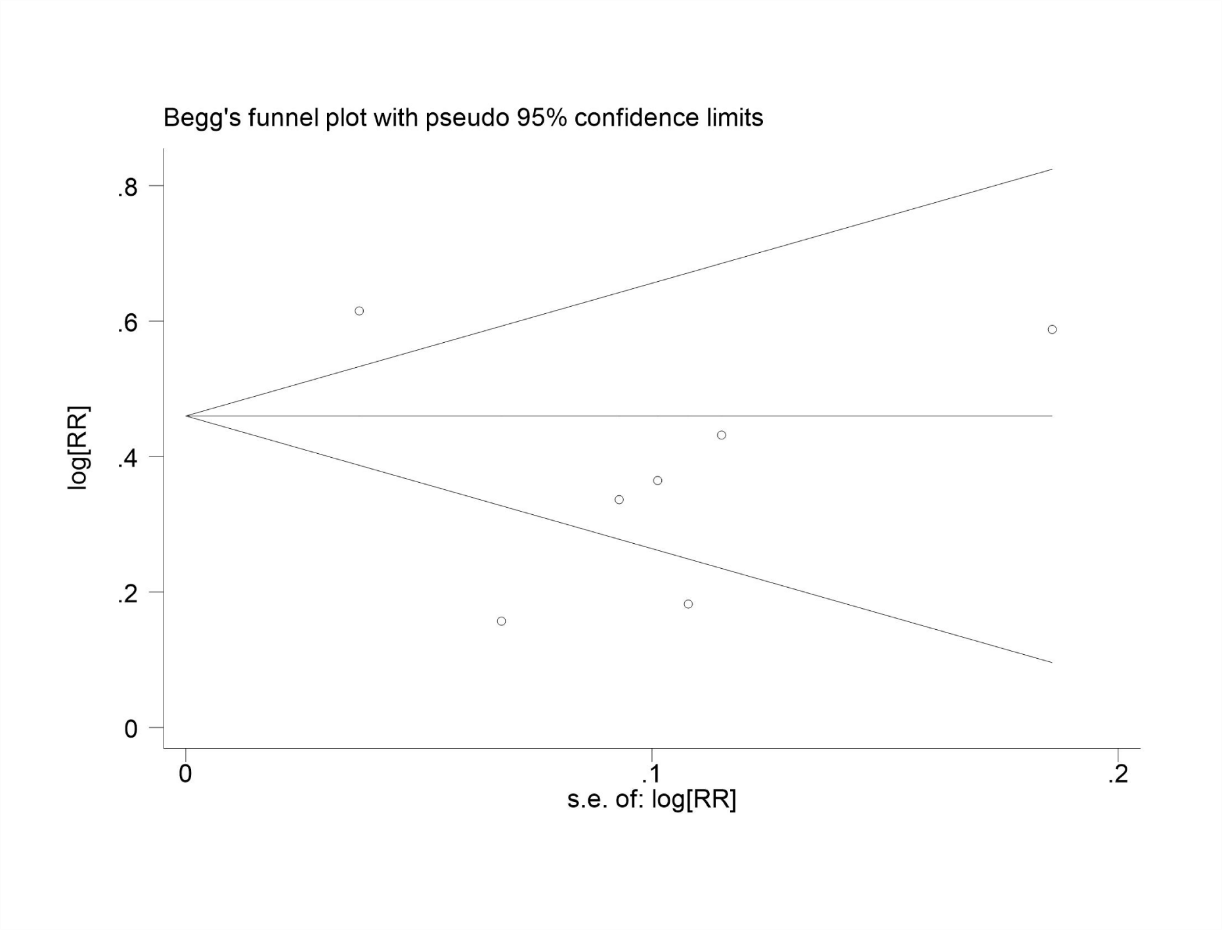
**

**
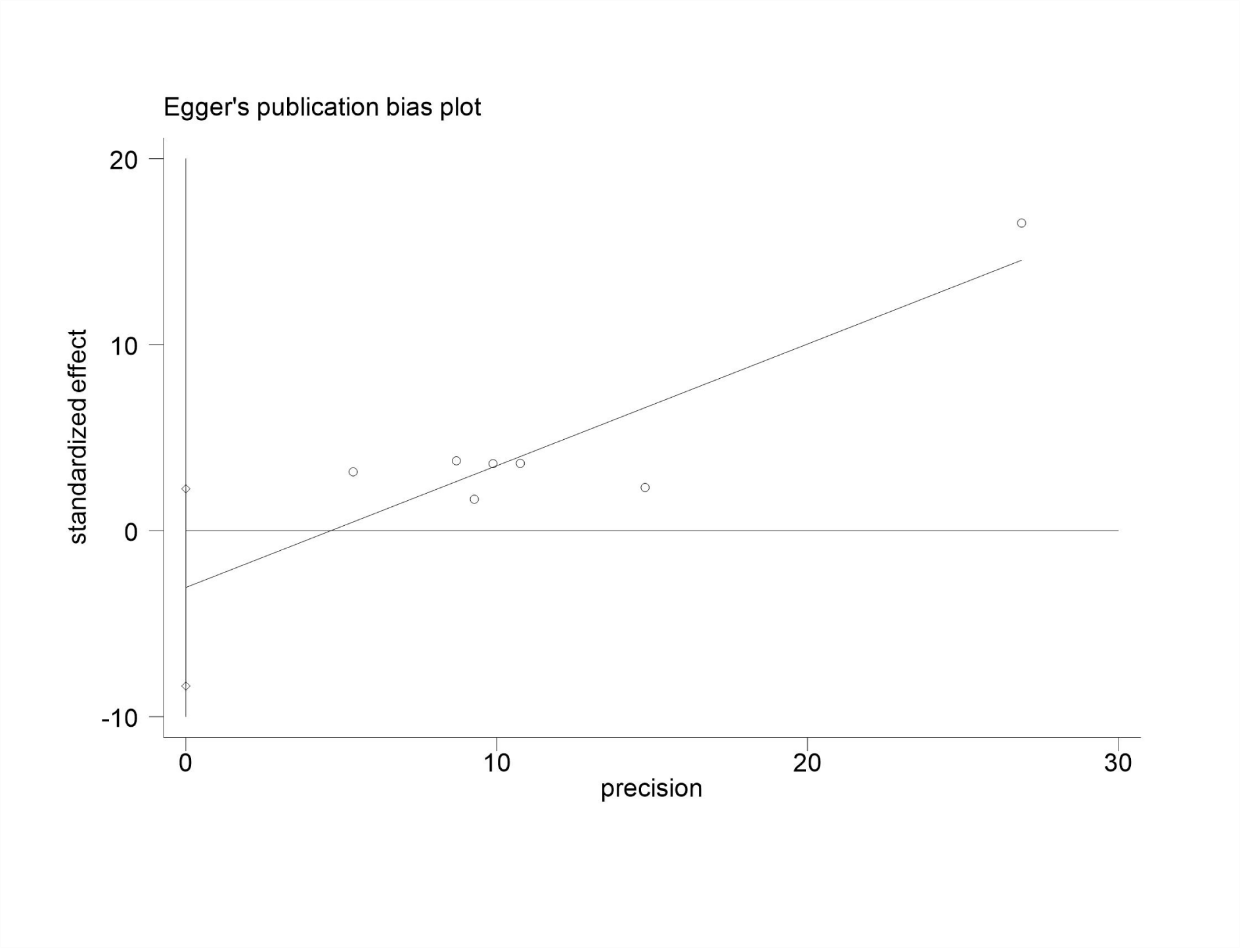
**

**5.2 *Hypophosphatemia vs Normophosphatemia. All-cause mortality.***

***
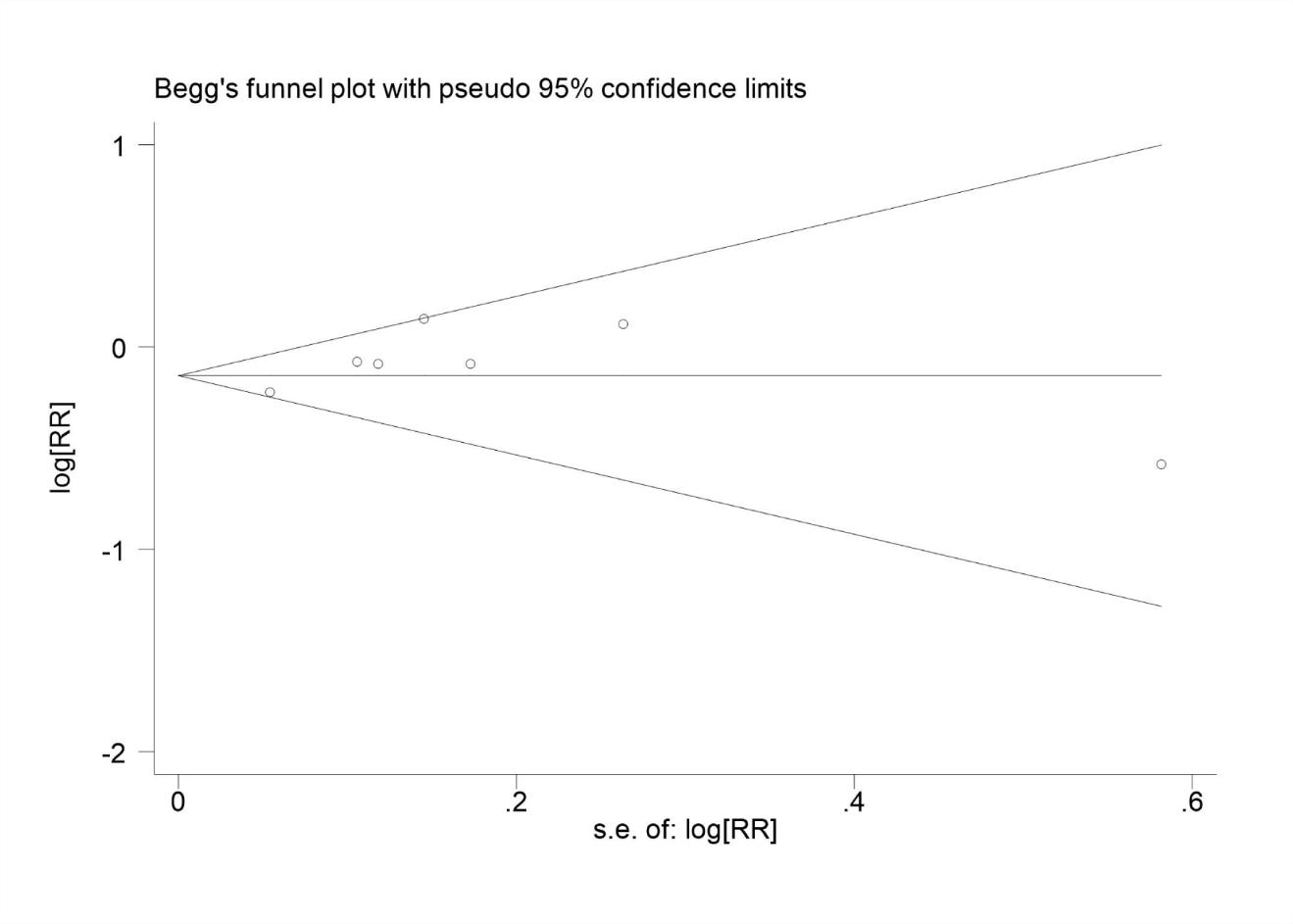
***

***
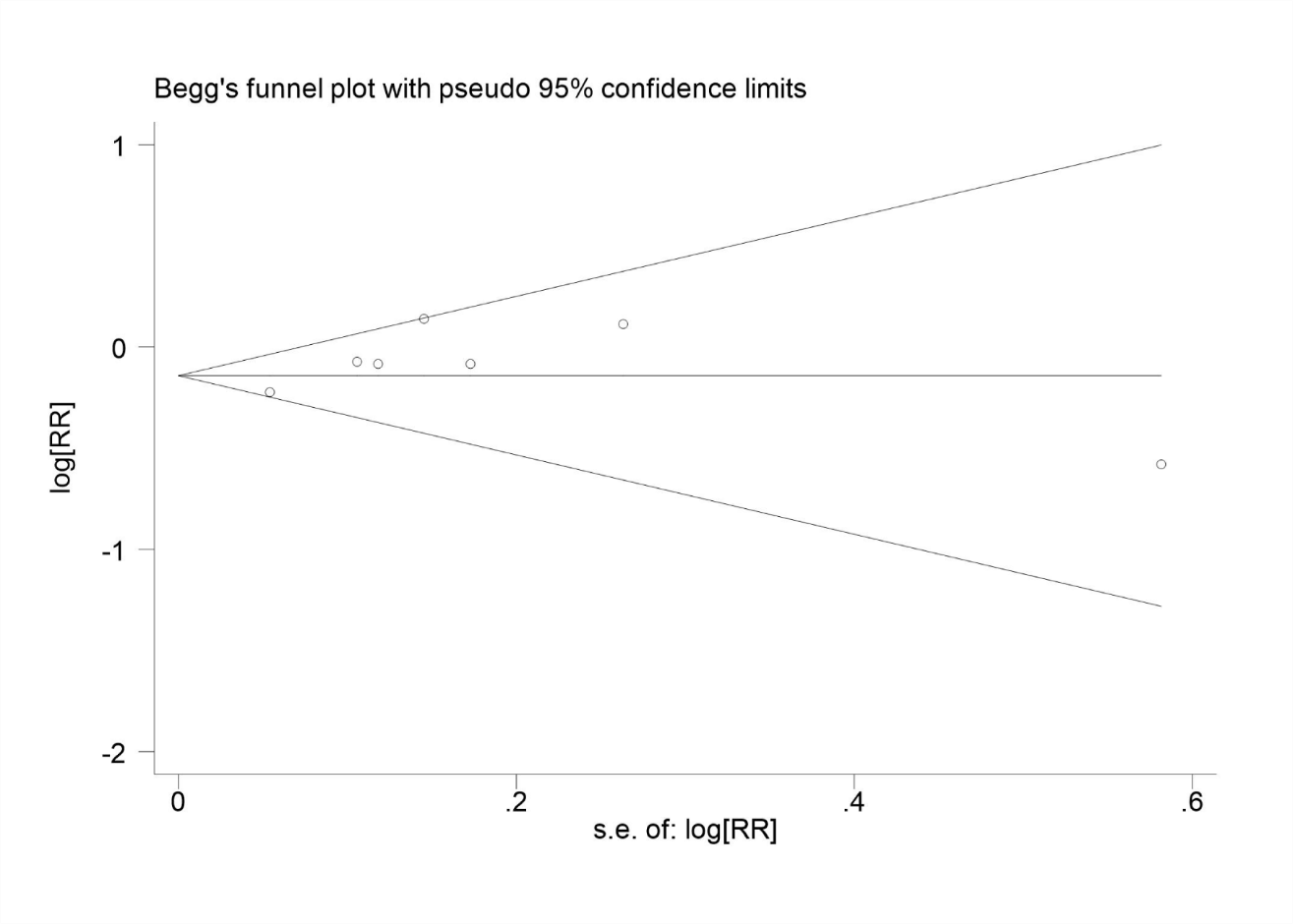
***

**5.3 *Per-increased.***

***
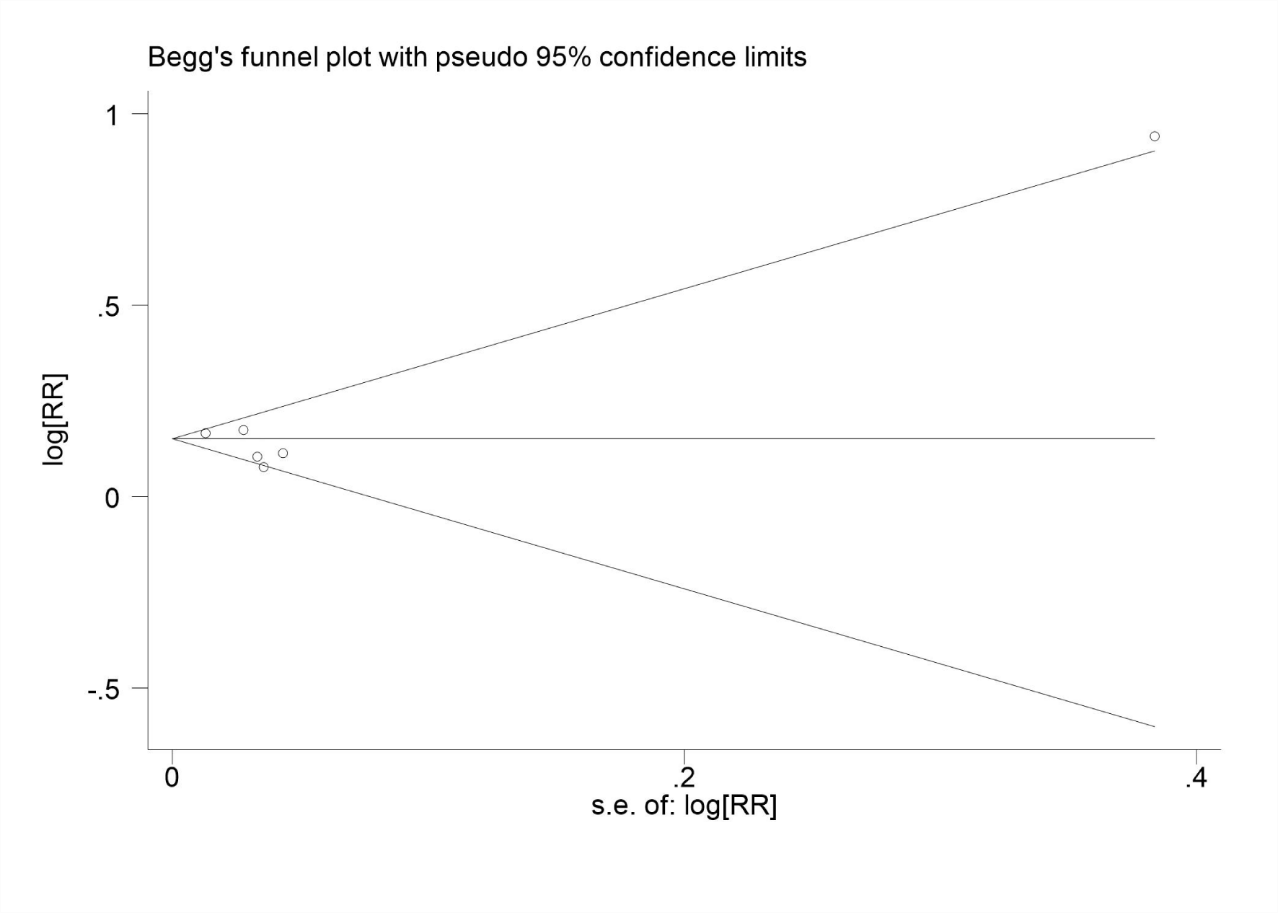
***

***
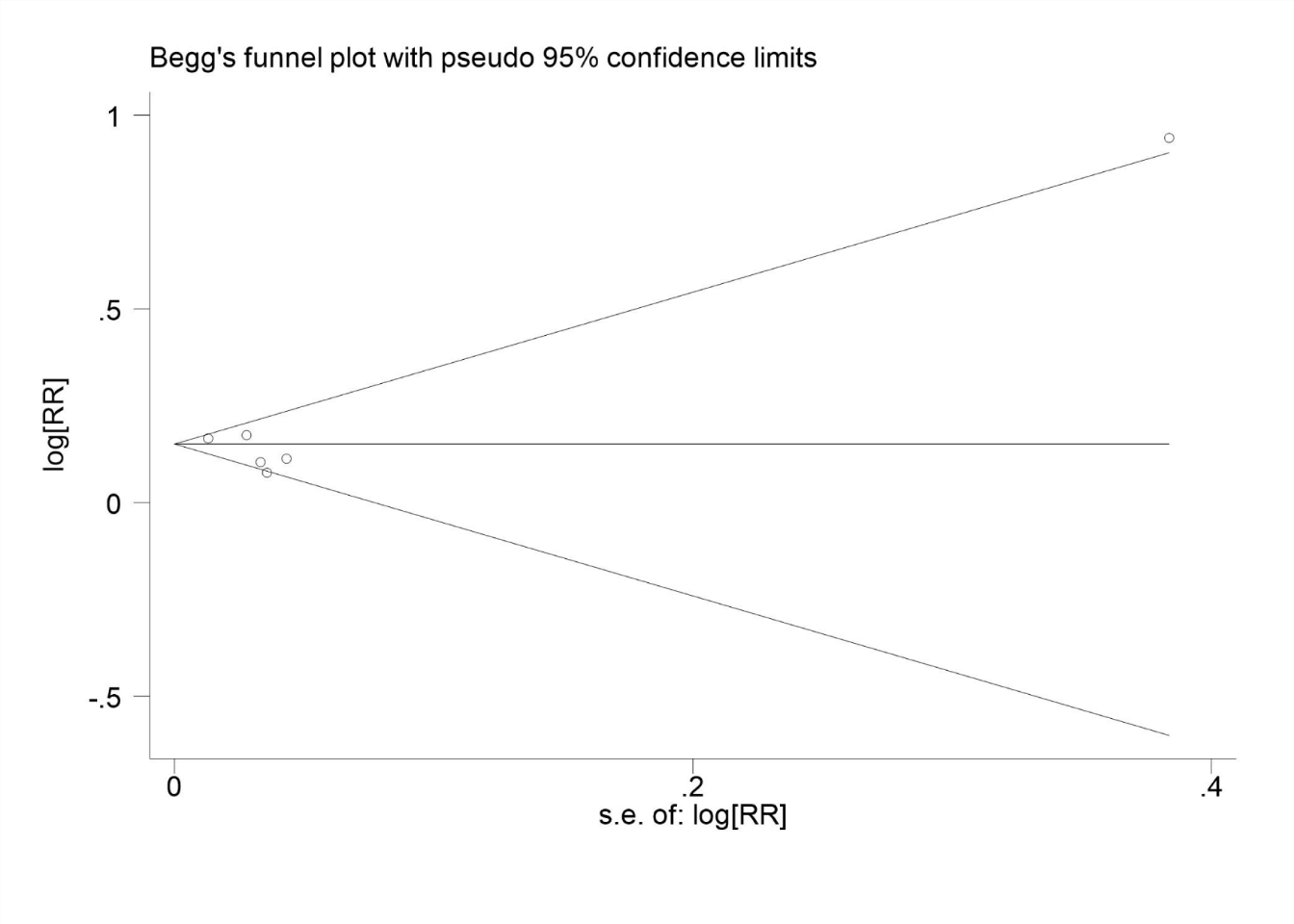
***

**6. Publication bias of secondary outcome.**

**6.1 *Hyperphosphatemia vs Normophosphatemia*. ICU LOS.**

**
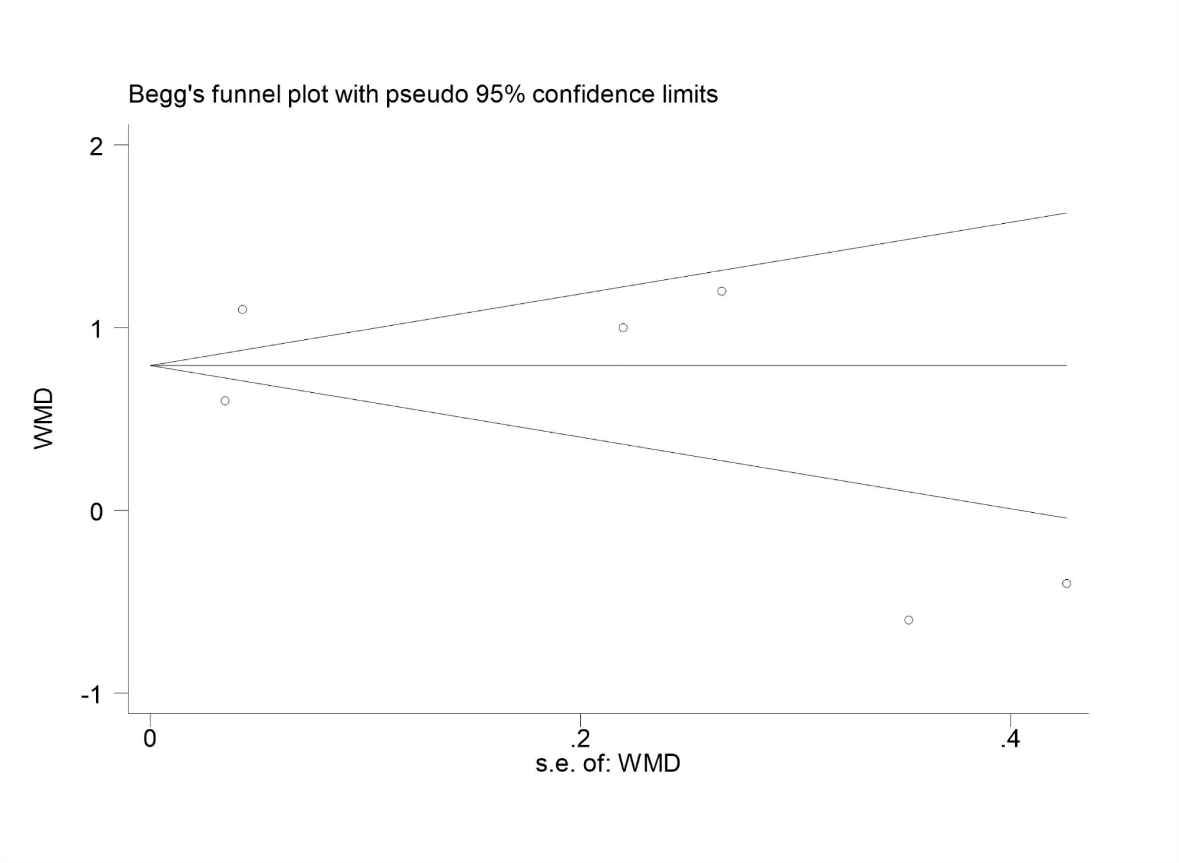
** **
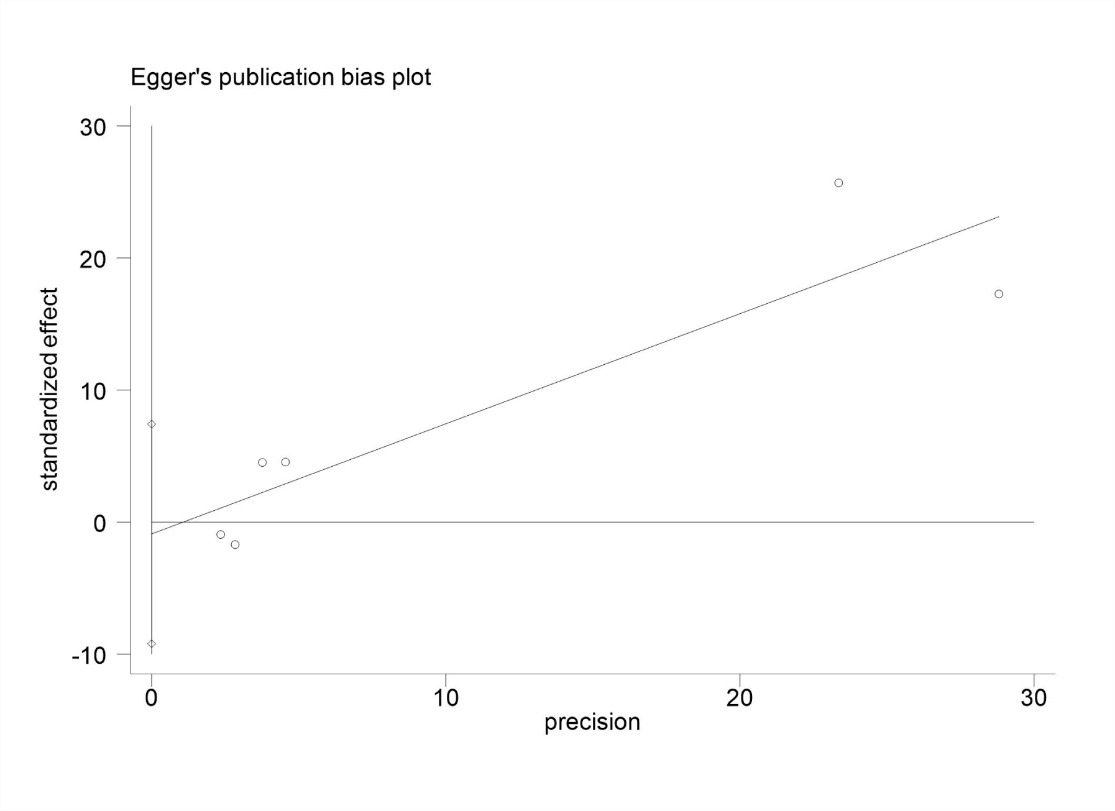
**5

**6.2 *Hypophosphatemia vs Normophosphatemia.* ICU LOS*.***

**
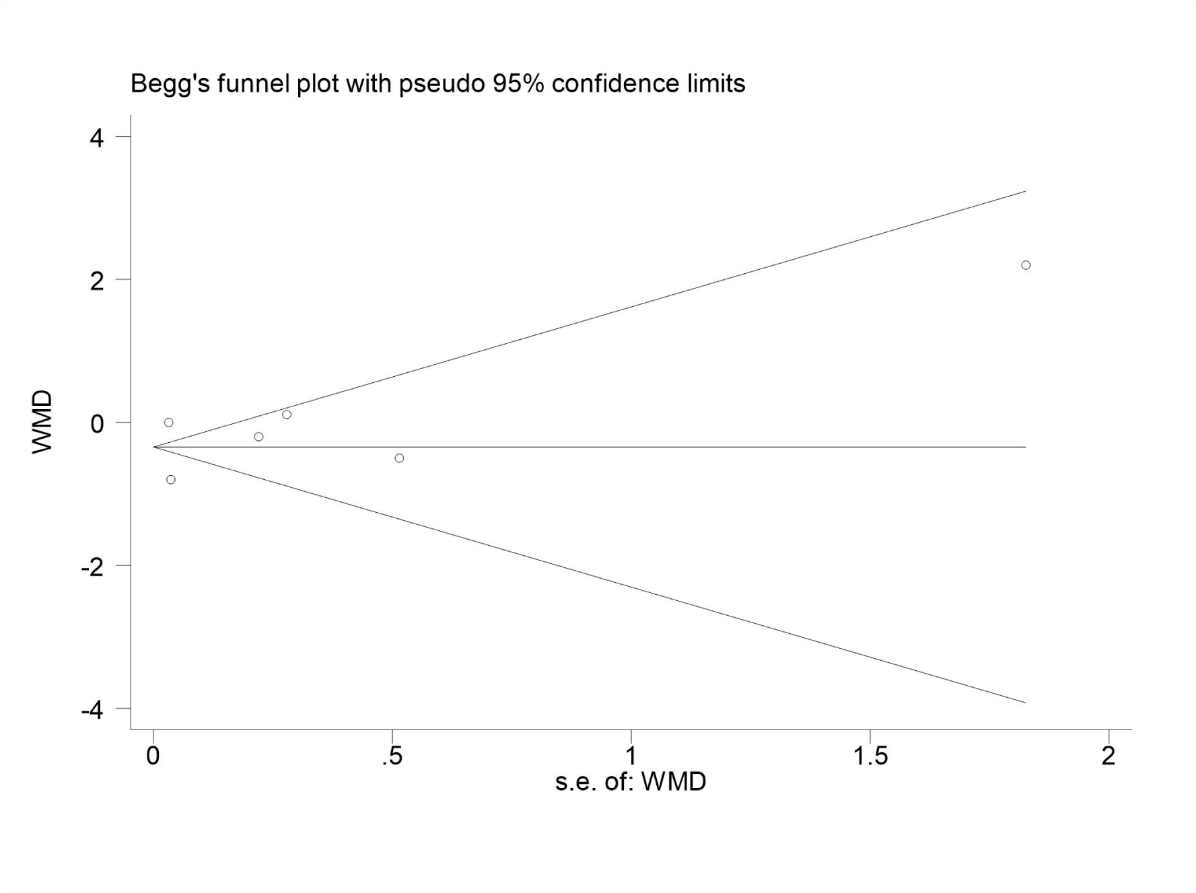
** **
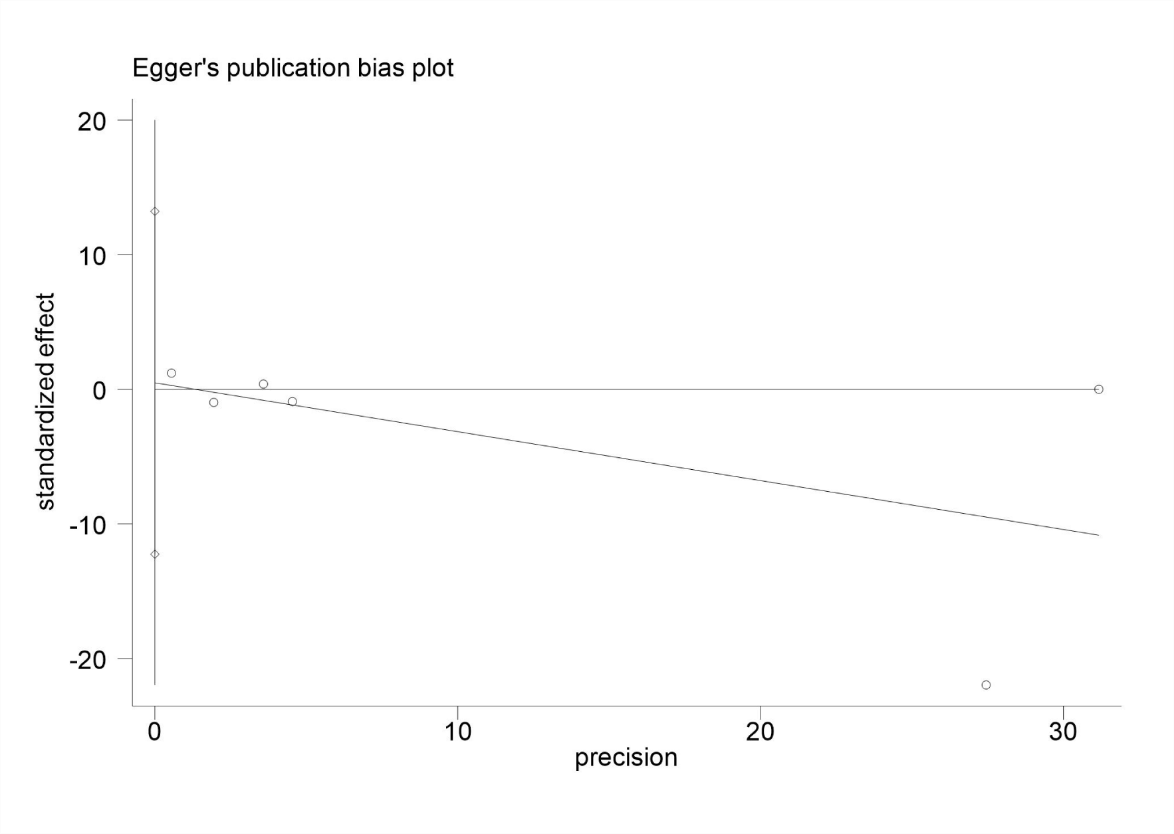
**

**6.3 *Hyperphosphatemia vs Normophosphatemia*. In-hospital LOS.**

**
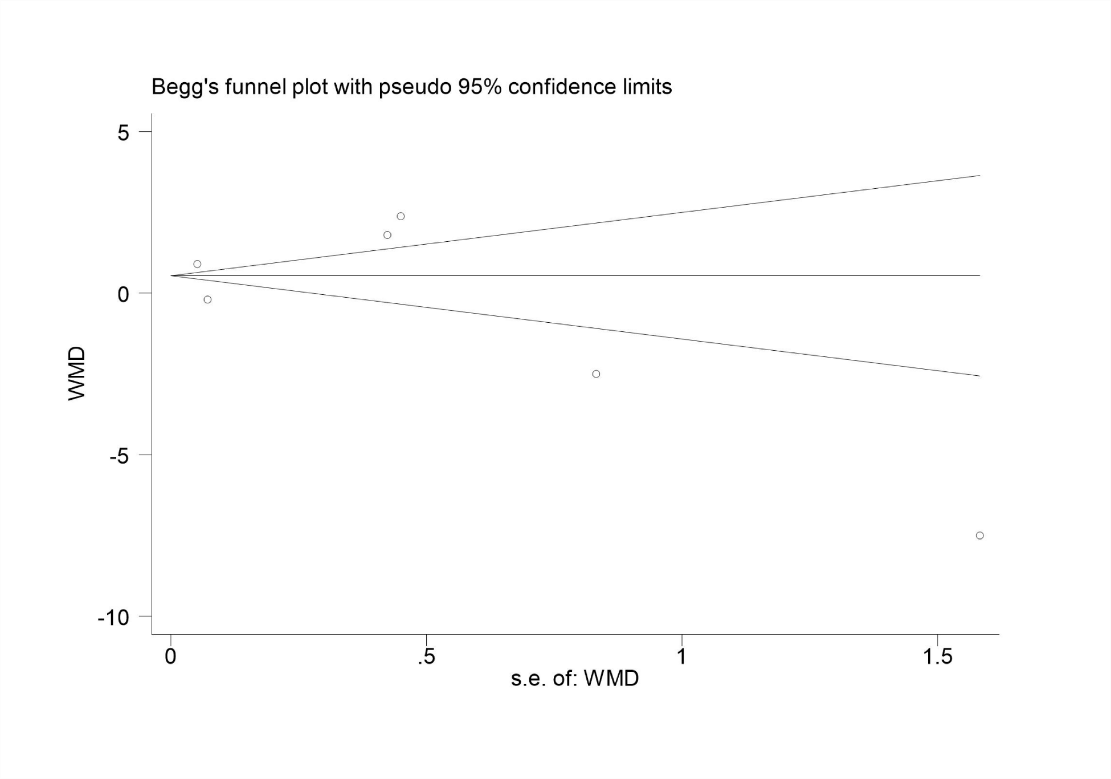
**

**
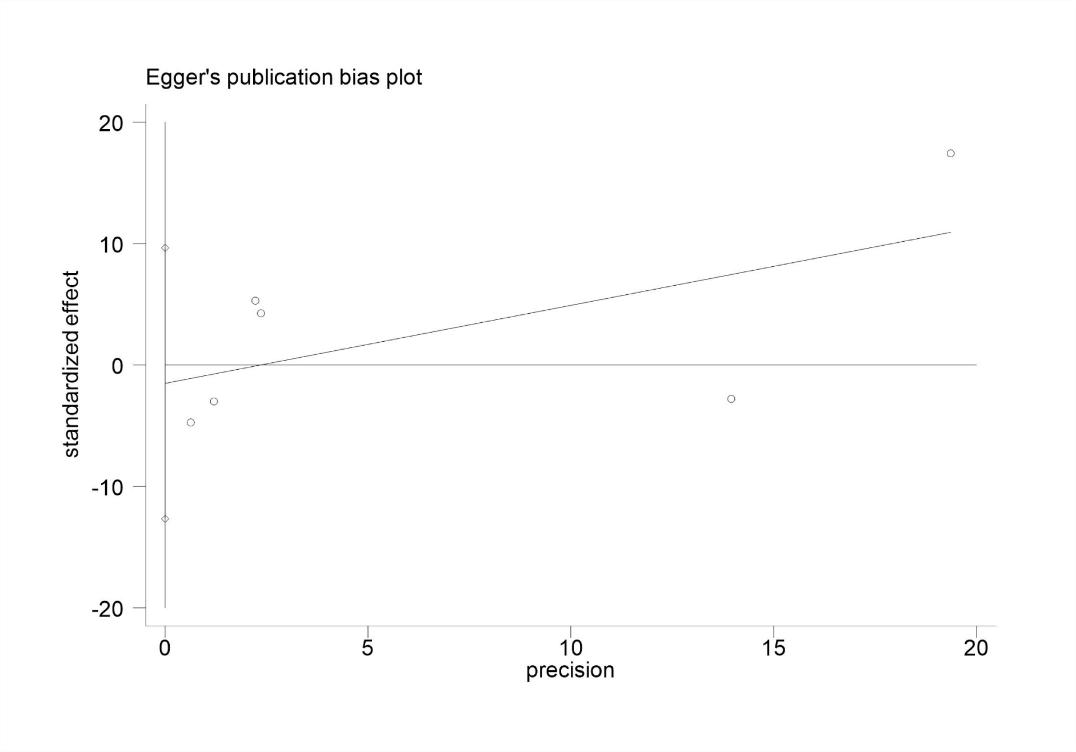
**

**6.4 *Hypophosphatemia vs Normophosphatemia.* In-hospital LOS*.***

**
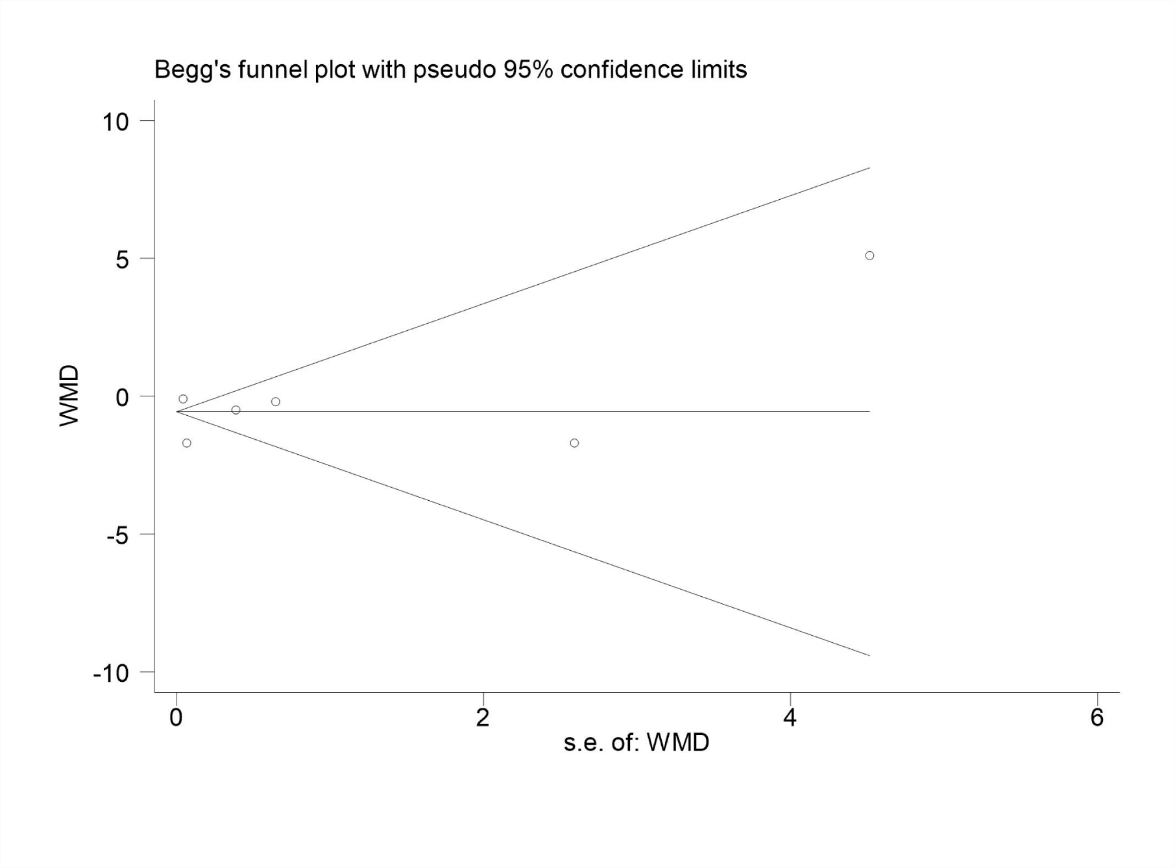
** **
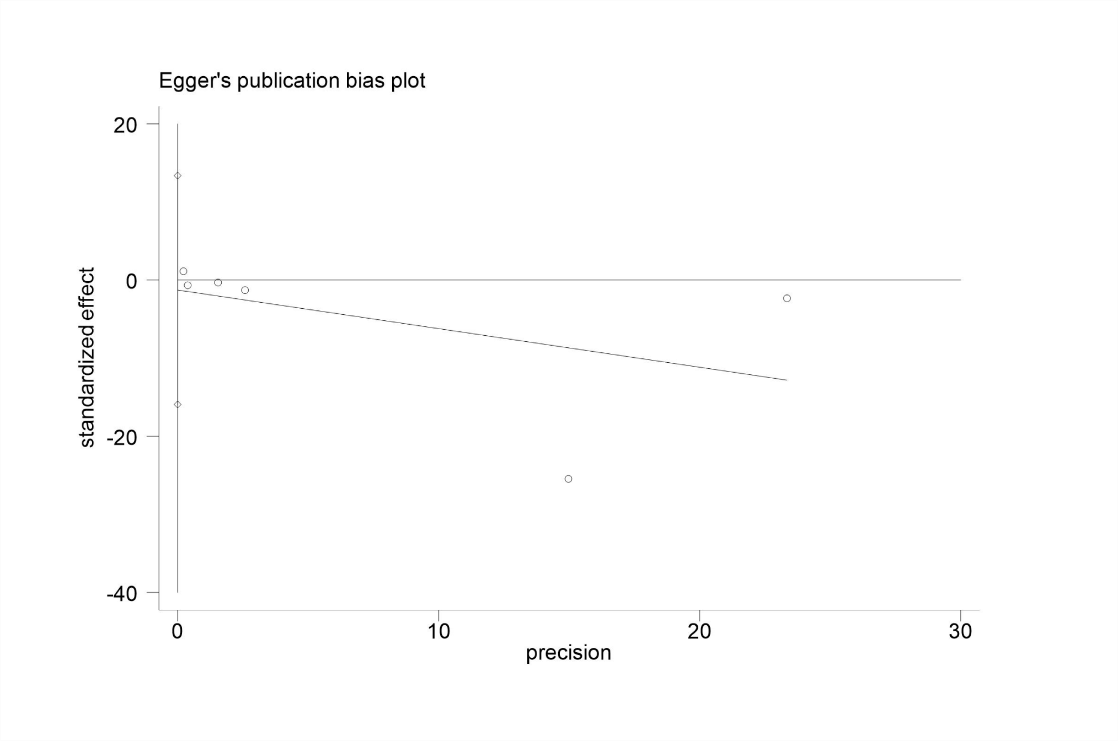
**
