# Supplementary material for: Prognostic value of serum phosphate levels in sepsis: a systematic review and meta-analysis
Source: PeerJ. 2023 Oct 13;11:e16241. doi: 10.7717/peerj.16241 (PMC10578301; doi:10.7717/peerj.16241)
Supplement: Supplemental Information 3 [file peerj-11-16241-s003.docx]

## Systematic Review and/or Meta-Analysis Rationale

For systematic reviews / meta-analyses, authors need to provide the following information:

**1.Comment:** The rationale for conducting the systematic review / meta-analysis;

**Responds:** The reliability of the early diagnosis of sepsis is declining due to the overlapping of early predictors with other disease symptoms, such as procalcitonin, C-reactive protein, lactic acid, etc., so it is necessary to explore new markers to timely diagnose and predict poor prognosis in sepsis. Phosphate, as an essential element in the human body, is beneficial to maintain muscle contraction and cell integrity, which also helps to transmit nerve stimulation and maintain normal organ function. At present, many studies have proved that phosphate level is related to the prognosis of sepsis, but the findings are conflicting and no relevant meta-analysis has been published. The purpose of our study is to summarize the research on this topic and elucidate the effects of different serum phosphate levels on sepsis, comprehensively explore its association with the prognosis of sepsis, and provide evidence for clinical practice.

**2.Comment:** The contribution that it makes to knowledge in light of previously published related reports, including other meta-analyses and systematic reviews.

**Responds:** A previous meta-analysis explored the association between high serum phosphate levels and all-cause mortality and ICU length of stay in critically ill patients. However, it focused only on critically ill patients with a wide range of diseases, lacked the exploration of the difference in prognosis of high phosphate levels in patients with different disease types and different pathogenesis, and only provided limited description of critically ill patients. In contrast, our study focuses on patients with sepsis, which to a certain extent reduces the impact of multiple diseases and factors on the stability of the results, and has a sufficient sample size for our statistics analysis, which also makes our research results more robust. We found that, compared with normal serum phosphate levels, high pre-intervention phosphate levels in sepsis patients were significantly associated with a higher risk of all-cause mortality, longer ICU length of stay, and possibly longer length of in-hospital stay, but the differences were not significant. Furthermore, elevated serum phosphate levels in patients with sepsis were significantly associated with an increased risk of mortality. Low phosphate levels in sepsis patients may reduce the incidence of adverse outcomes, but the difference was not significant.
